# Neratinib plus trastuzumab is superior to pertuzumab plus trastuzumab in HER2-positive breast cancer xenograft models

**DOI:** 10.1038/s41523-021-00274-0

**Published:** 2021-05-27

**Authors:** Jamunarani Veeraraghavan, Carolina Gutierrez, Vidyalakshmi Sethunath, Sepideh Mehravaran, Mario Giuliano, Martin J. Shea, Tamika Mitchell, Tao Wang, Sarmistha Nanda, Resel Pereira, Robert Davis, Kristina Goutsouliak, Lanfang Qin, Carmine De Angelis, Irmina Diala, Alshad S. Lalani, Chandandeep Nagi, Susan G. Hilsenbeck, Mothaffar F. Rimawi, C. Kent Osborne, Rachel Schiff

**Affiliations:** 1 Lester and Sue Smith Breast Center, Baylor College of Medicine, Houston, TX USA; 2 Dan L Duncan Comprehensive Cancer Center, Baylor College of Medicine, Houston, TX USA; 3 Department of Medicine, Baylor College of Medicine, Houston, TX USA; 4 Department of Pathology, Baylor College of Medicine, Houston, TX USA; 5 Department of Biochemistry and Molecular Biology, Baylor College of Medicine, Houston, TX USA; 6 Department of Clinical Medicine and Surgery, University of Naples Federico II, Naples, Italy; 7 Puma Biotechnology Inc., Los Angeles, CA USA; 8 Department of Molecular and Cellular Biology, Baylor College of Medicine, Houston, TX USA

**Keywords:** Breast cancer, Targeted therapies

## Abstract

Lapatinib (L) plus trastuzumab (T), with endocrine therapy for estrogen receptor (ER)+ tumors, but without chemotherapy, yielded meaningful response in HER2+ breast cancer (BC) neoadjuvant trials. The irreversible/pan-HER inhibitor neratinib (N) has proven more potent than L. However, the efficacy of N+T in comparison to pertuzumab (P) + T or L + T (without chemotherapy) remains less studied. To address this, mice bearing HER2+ BT474-AZ (ER+) cell and BCM-3963 patient-derived BC xenografts were randomized to vehicle, N, T, P, N+T, or P+T, with simultaneous estrogen deprivation for BT474-AZ. Time to tumor regression/progression and incidence/time to complete response (CR) were determined. Changes in key HER pathway and proliferative markers were assessed by immunohistochemistry and western blot of short-term-treated tumors. In the BT474-AZ model, while all N, P, T, N + T, and P + T treated tumors regressed, N + T-treated tumors regressed faster than P, T, and P + T. Further, N + T was superior to N and T alone in accelerating CR. In the BCM-3963 model, which was refractory to T, P, and P + T, while N and N + T yielded 100% CR, N + T accelerated the CR compared to N. Ki67, phosphorylated (p) AKT, pS6, and pERK levels were largely inhibited by N and N + T, but not by T, P, or P + T. Phosphorylated HER receptor levels were also markedly inhibited by N and N + T, but not by P + T or L + T. Our findings establish the efficacy of combining N with T and support clinical testing to investigate the efficacy of N + T with or without chemotherapy in the neoadjuvant setting for HER2+ BC.

## Introduction

The human epidermal growth factor receptor (HER2)-positive (+) subtype, which accounts for 15–20% of all breast cancers, is defined by HER2 (*ERBB2*) gene amplification and/or protein overexpression. HER2 is a key member of the HER family that also includes HER1/EGFR, HER3, and HER4. While multiple ligands activate the HER1, 3, and 4 receptors, the ligand-less HER2 is activated via heterodimerization with other ligand-bound HER receptors or by homodimerization in tumors overexpressing HER2. The activated receptors govern an intricate signaling cascade (e.g., PI3K/AKT, RAS/MAPK (ERK) pathways), which regulates multiple malignant cellular processes including proliferation, survival, and metastasis^1,2^. By virtue of recent development of potent HER2-targeted agents, patients with HER2+ breast cancer have witnessed marked improvements in outcome. The humanized anti-HER2 monoclonal antibody (mAb) trastuzumab (T) led the way to be the first FDA-approved biologic agent for HER2+ breast cancer, in combination with chemotherapy. Together with pertuzumab (P), another anti-HER2 mAb, the two antibodies act complementarily to inhibit HER2 signaling, while also eliciting antibody dependent cell-mediated cytotoxicity (ADCC) via engagement of the host immune system^1,3–5^. In addition, other classes of HER2-targeted agents including the small-molecule tyrosine kinase inhibitors (TKI) (e.g., lapatinib (L), a dual HER1/2 TKI), and the antibody drug conjugates (ADC) (e.g., trastuzumab emtansine (T-DM1)) composed of the mAb T linked to a cytotoxic agent, have demonstrated clinical efficacy.

Given the redundancy in HER family signaling, mounting evidence implicate the need to comprehensively block the entire HER receptor layer to ensure maximum anti-tumor efficacy and to prevent resistance mediated by the unblocked HER receptors. We and others have previously shown that combinatorial HER2 blockade, through simultaneous use of two or more anti-HER2 agents, yields anti-tumor efficacy superior to single agents^6–8^ in various xenograft models. Further, in ER+/HER2+ breast xenograft models complete tumor eradication depended on concurrent blockade of ER together with anti-HER2 therapy^6–8^, since uninhibited ER can transmit alternative survival and proliferative signals to evade HER2 blockade. This notion has also been echoed clinically in multiple trials in the neoadjuvant, adjuvant, and metastatic settings, except in the B-52 trial in which while there was a numerical increase in pathologic complete response (pCR) rates with endocrine deprivation, the improvement was not statistically significant^9–12^. Specifically, T in combination with L or P, plus endocrine therapy for ER+ tumors in some trials, demonstrated superior anti-tumor efficacy compared to single agents, although most clinical trials also included chemotherapy^9,13–17^.

With the field of cancer therapy headed towards personalized treatment, efforts are increasingly being focused on de-escalating therapy in order to reduce or eliminate chemotherapy-associated toxicity due to unwarranted escalation of treatment for patients who could otherwise be spared chemotherapy or could benefit from less chemotherapy, without compromising outcomes. In our recently reported chemotherapy-sparing neoadjuvant trials of HER2+ breast cancer, we showed that a subset of patients with large tumors (~25–30%) achieve pCR with dual L + T therapy alone, without chemotherapy^15–18^. The recent development and approval of next-generation HER2-targeted agents, including irreversible pan-HER TKIs that are more potent than L, has further enriched the arsenal of anti-HER2 agents. If used in combination with T to completely and complementarily block the HER receptor layer via different mechanisms of action, these small-molecule targeted agents hold the potential to improve treatment efficacy, in both the primary and micro-metastatic niches, especially brain, and to improve the outcome of patients with HER2-amplified and addicted tumors, without chemotherapy^19^.

The irreversible pan-HER inhibitor neratinib (N), which covalently binds to the kinase domain of HER1/EGFR, HER2, and HER4, has shown clinical promise leading to its FDA approval for extended treatment of early-stage HER2+ breast cancer in the adjuvant setting following T-based therapy. N was also recently FDA-approved in the metastatic setting in combination with capecitabine. Moreover, the efficacy of N, either alone or in combination with other targeted therapies, in different clinical settings is being evaluated in several ongoing clinical trials. However, the therapeutic efficacy of N in combination with T (N + T), and how it compares to P + T or L + T, particularly in the absence of chemotherapy, has not been fully explored. We hypothesize that dual HER2 inhibition using N + T will prove more effective than single-agent treatments, and equally potent or more effective than P + T or L + T, in achieving a more complete blockade of the HER signaling and in inhibiting tumor growth. In this study, we sought to evaluate the therapeutic benefit of N + T and compare its efficacy to P + T using HER2+ breast cancer xenograft models.

## Results

### Neratinib-containing anti-HER2 regimen is superior to trastuzumab and pertuzumab in BT474 cell-derived xenograft model

**Fig. 1:**
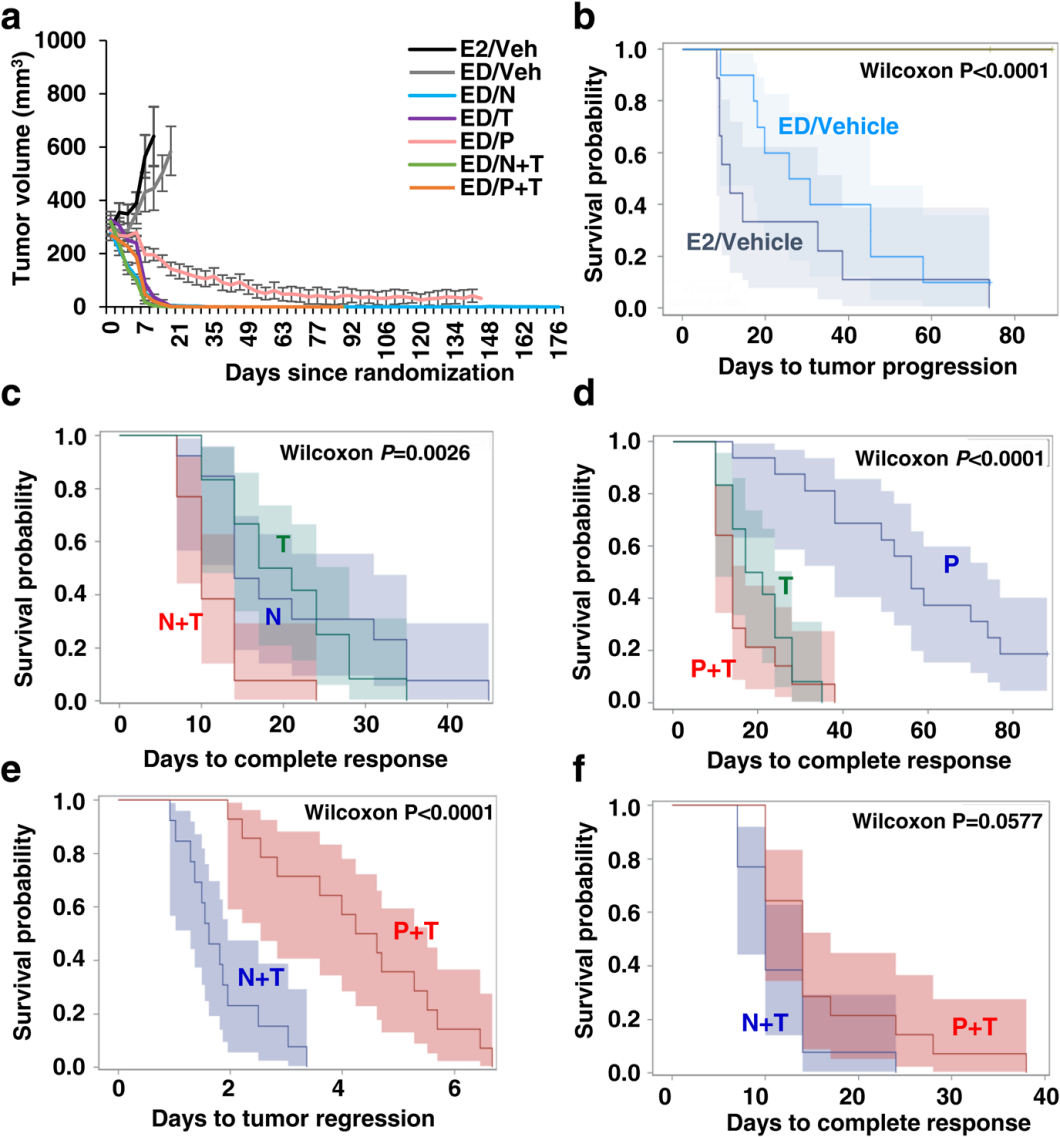
Neratinib (N) containing anti-HER2 regimen shows superior anti-tumor efficacy than trastuzumab (T) and pertuzumab (P) in BT474 cell-derived xenograft model. **a** Average growth curves of tumors treated with Vehicle, N, T, P, N + T, or P + T (*n* = 9–14). Results are presented as mean tumor volume ± SEM. Kaplan–Meier analysis of **b** time to tumor progression in mice treated with different anti-HER2 therapies, **c** time to complete response in mice treated with N, T, or N + T, **d** time to complete response in mice treated with T, P, or T + P, and **e** time to tumor regression and **f** time to complete response in mice treated with N + T or P + T. Shaded areas represent point-wise 95% confidence regions, calculated using the loglog transform. *P* values are indicated on plots, Wilcoxon test; Veh Vehicle, ED estrogen deprivation.

To evaluate the efficacy of different anti-HER2 treatment regimens, we first used the ER+/HER2-amplified BT474/AZ cells with high and homogeneous levels of HER2, hereafter referred to as BT474 (Supplementary Fig. 1). BT474/AZ is a subline of the BT474/ATCC cell line that belongs to the HER2-enriched subtype^20^ and can grow effectively as xenografts in vivo, as we previously reported^6–8,21^. Mice bearing BT474 xenografts, established with exogenous supplementation of estrogen (E2), were randomized to either vehicle ± estrogen deprivation (ED), or N, T, P, N + T, and P + T, all under ED to mimic aromatase inhibitor treatment in post-menopausal patients (Supplementary Fig. 1). Mice treated with E2+ vehicle and ED+ vehicle showed steady tumor growth, with a median time to tumor progression (TTP) of 8 and 25 days, respectively (Fig. 1a, b and Table 1). Conversely, 100% tumor regression was observed in the N, T, P, N + T, and P + T arms (Supplementary Fig. 2).

**Table 1:** Primary study endpoints following different anti-HER2 therapies in BT474 cell line-derived and BCM-3963 patient-derived xenograft models.

Model/treatment	No. of mice	Median TTP days (95% CI)	Median TTR days (95% CI)	Median TCR days (95% CI)	CR (%)
**BT474-AZ model**					
E2 + Vehicle	9	8 (4.58–35.36)	–	–	0
ED + Vehicle	10	25 (5.85–42.2)	–	–	0
ED + N	13	–	2 (1.68–2.43)	14 (14.00–31.00)	100
ED + T	12	–	5 (2.64–6.44)	19 (10.00–28.00)	100
ED + P	12	–	18 (6.93–49.01)	54 (24.00–NA)	92
ED + N + T	13	–	2 (1.29–1.95)	10 (7.00–14.00)	100
ED + P + T	14	–	4 (2.54–5.51)	14 (14.00–31.00)	100
**BCM-3963 model**					
Vehicle	15	11 (7.57–17.29)	–	–	0
N	15	–	4 (2.52–7.10)	17 (14.00–28.00)	100
T	14	16 (6.52–22.59)	–	–	0
P	13	19 (10.89–25.29)	–	–	0
N + T	19	–	6 (3.22–8.31)	14 (14.00–17.00)	100
P + T	16	17 (13.61–22.14)	–	–	0

*E2* estrogen, *ED* estrogen deprivation, *N* neratinib, *T* trastuzumab, *P* pertuzumab, *TTP* time to tumor progression, *TTR* time to tumor regression, *TCR* time to complete response, *CR* complete response, *CI* confidence interval.

Tumors treated with N alone regressed faster, with a median time to tumor regression (TTR) of 2 days, compared to tumors treated with T (5 days, *P* = 0.1404) or P (18 days, *P* < 0.001) alone (Table 1). While not significant, a similar trend was also observed with complete response (CR), with the N-treated group achieving CR in a median time of 14 days compared to 19 and 54 days in the T (*P* = 1.000) and P (*P* = 0.1617) groups, respectively. Importantly, tumors treated with N + T regressed slightly faster than those treated with P (*P* < 0.0001), T (*P* = 0.0032), or P + T (*P* = 0.0327), and the combination was also slightly but significantly superior to N (*P* = 0.0182) or T (*P* = 0.0074) alone in accelerating CR (Fig. 1c). As a single agent, T proved superior to P in achieving CR (Fig. 1d, *P* = 0.0007) but not tumor regression (*P* = 0.7601). Further, while P + T was significantly better than P alone (*P* < 0.0001), the combination did not outperform T alone (*P* = 0.4335) in accelerating CR. A head-to-head comparison of the combination treatments showed that N + T offered substantial benefit over P + T in accelerating tumor regression (*P* < 0.0001) (Fig. 1e), with a similar trend for CR, though it did not reach statistical significance (*P* = 0.0577) (Fig. 1f). Overall, our results highlight the therapeutic superiority of N, both alone and, even further, in combination with T, over P + T in a HER2-amplified cell-derived xenograft model.

Additionally, the anti-HER2 treatment regimens were also evaluated for their potential to achieve tumor eradication. When there was no palpable tumor for ~120 days on-treatment, treatments were stopped in the N (*n* = 13), T (*n* = 8), P (*n* = 11), N + T (*n* = 12), and P + T (*n* = 13) arms and mice were maintained under continued ED for 2 weeks after which some mice in the N (*n* = 5), T (*n* = 2), N + T (*n* = 5), and P + T (*n* = 7) arms were further randomized to E2 supplementation. Mice in both E2 and ED groups were monitored for ~60 days for recurrence. Importantly, no recurrence was observed in the T, N + T, and P + T arms in both the E2 and ED group. In the N alone arm, one mouse each in the E2 supplementation and ED groups showed tumor recurrence (Supplementary Fig. 2). Notably, no significant changes in body weight were observed in the vehicle and anti-HER2 drug-treated groups, attesting to the overall safety and tolerability of the anti-HER2 regimens tested (Supplementary Fig. 2).

### Neratinib-containing anti-HER2 regimens are effective in a patient-derived xenograft model that is refractory to trastuzumab and pertuzumab

**Fig. 2:**
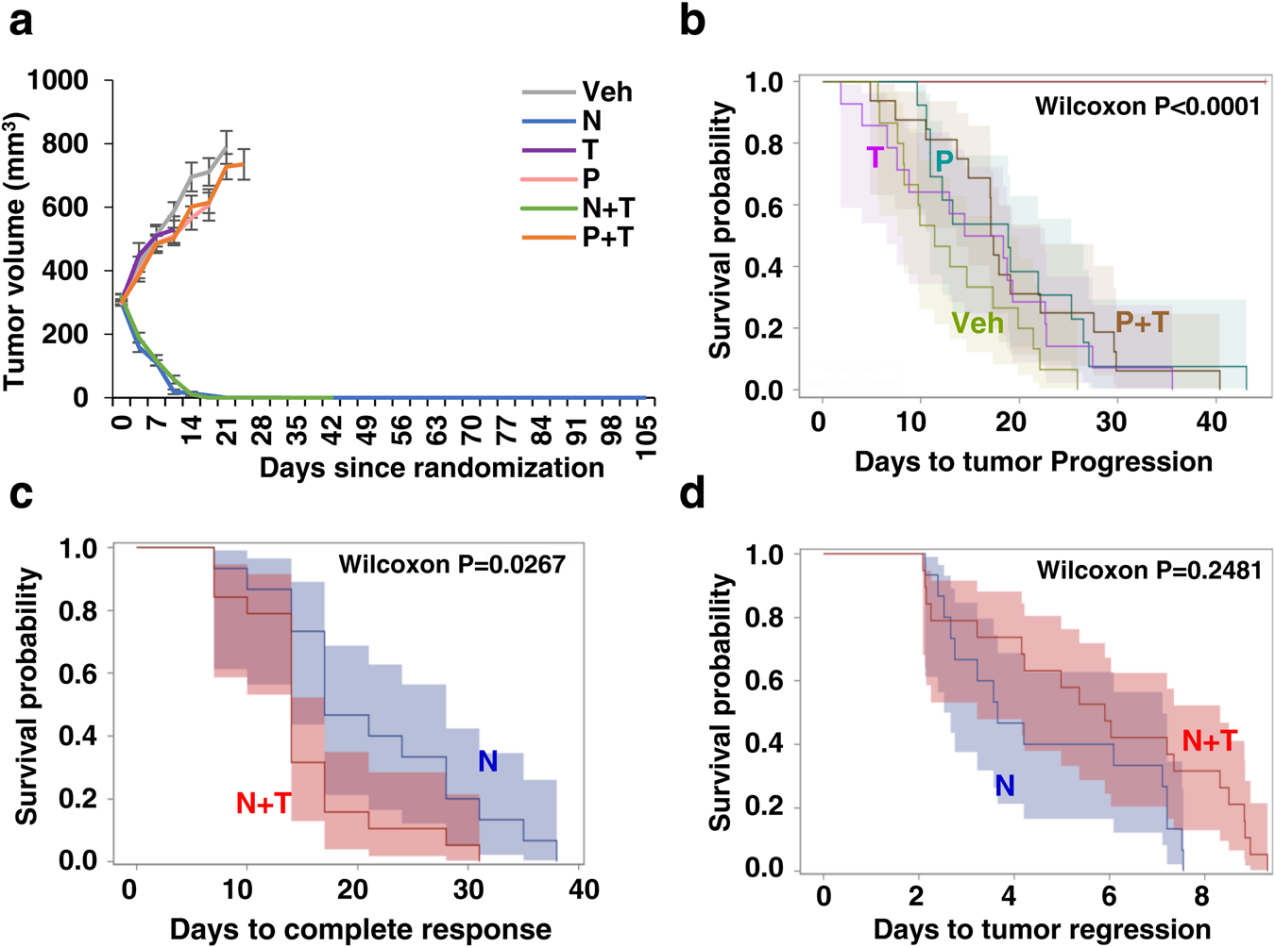
Neratinib (N) containing anti-HER2 regimens effectively achieve tumor regression and eradication in a patient-derived xenograft model that is refractory to trastuzumab (T) and pertuzumab (P). **a** Average growth curves of tumors treated with Vehicle, N, T, P, N + T, or P + T (*n* = 13–19). Results are presented as mean tumor volume ± SEM. Kaplan–Meier analysis of **b** time to tumor progression in mice treated with different anti-HER2 therapies, and **c** time to complete response and **d** time to tumor regression in mice treated with N or N + T. Shaded areas represent point-wise 95% confidence regions, calculated using the loglog transform. *P* values are indicated on plots, Wilcoxon test. Veh Vehicle.

To corroborate our findings from the BT474 xenograft model, we extended our investigation of the efficacy of Vehicle, N, T, P, N + T, or P + T regimens to BCM-3963, an ER−/HER2+ patient-derived breast cancer xenograft (PDX) model of the HER2-enriched subtype^22^ (Supplementary Fig. 1). This PDX model was developed using a pre-treatment tumor biopsy from a patient who responded to neoadjuvant lapatinib (L) and T. Unlike the BT474 tumors, the BCM-3963 tumors were completely refractory to T and P, both when used as a single agent and when combined together (Fig. 2a, b). Interestingly, however, these tumors remained highly sensitive to both single-agent N and to the combination of N with T, with a median TTR of 4 and 6 days, respectively (Supplementary Fig. 3 and Table 1). While the addition of T to N did not offer added benefit compared to N alone (*P* = 0.2481) in achieving tumor regression (Fig. 2d), the combination was slightly but significantly better than single-agent N in accelerating CR (*P* = 0.0267), with a median time to complete response (TCR) of 14 vs 17 days (Fig. 2c and Table 1). Our findings thus suggest that, while T, by itself, did not have an apparent therapeutic benefit in this PDX model, when combined with another anti-HER2 agent, particularly TKI, it significantly enhanced the efficacy of the TKI, N in our case. In validation of this notion, we have previously shown that with L + T treatment all the BCM-3963 tumors achieved complete or near-complete tumor regression, mimicking the clinical course of the patient from whom the tumor originated^22^. Importantly, however, when mice were treated with L alone only partial response was observed with either stable disease or partial tumor regression, and many tumors progressed later after an initial transient regression (Supplementary Fig. 4 and Table 1).

To evaluate tumor eradication, treatment was stopped in the N (*n* = 15) and N + T (*n* = 16) arms when there was no palpable tumor for ~120 days on-treatment, and mice were monitored for additional ~60 days for tumor recurrence. Notably, no recurrence was observed in both the N and N + T arms. Further, similar to our observation in the BT474 model, no significant changes in body weight were observed in the vehicle and the anti-HER2 treated groups, suggesting that the anti-HER2 regimens were well tolerated (Supplementary Fig. 3).

### Neratinib-containing regimens are more effective in inhibiting the HER signaling pathway and tumor cell proliferation in BT474 HER2+ cell-derived xenograft model

**Fig. 3:**
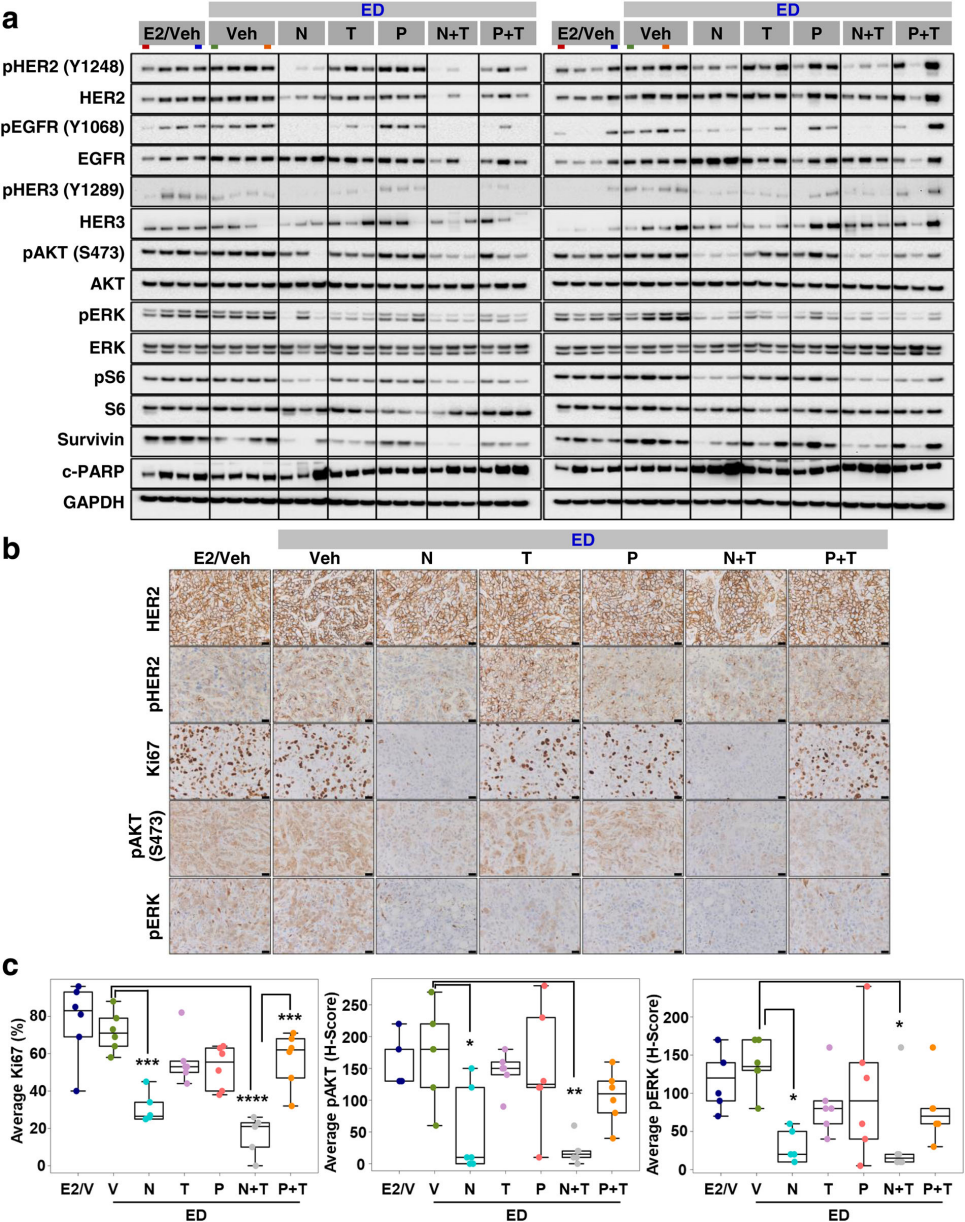
Neratinib-containing regimens significantly inhibit tumor cell proliferation and HER2 signaling in short-term treated BT474-AZ xenograft tumors. **a** Western blot analyses of alterations in level and activation of proteins along the HER signaling axis. Each of the seven short-term treatment arms had six tumors, which were run in two separate gels with each gel containing three tumors per arm. Colored bars below the treatment arms in gray boxes denote duplicate samples loaded as comparison control between the left and right blots. **b** Representative HER2, pHER2, Ki67, pAKT, and pP44/42 ERK immunohistochemical staining. **c** Box plots showing average Ki67 (%), pAKT (*H*-Score), and pERK (*H*-Score) protein levels by immunohistochemistry. Box plots indicate median and interquartile range (IQR). The lower and upper hinges correspond to the first (25th percentile) and third (75th percentile) quartiles, respectively. The whiskers extend to about 1.5 × IQR from the hinge, and data points beyond the whiskers are suspected outliers. E2 estrogen, ED estrogen deprivation, Veh/V vehicle, N neratinib, T trastuzumab, P pertuzumab, IHC images in **b** are of ×40 magnification, Scale bar: 50 μm. *P < 0.05, **P < 0.01, ***P < 0.001, ****P < 0.0001.

To better understand the mechanism of action of the HER2-targeted treatments, BT474 tumors (*n* = 6) treated for 3.5 days with either vehicle, N, T, P, N + T, or P + T were subjected to molecular/signaling analyses. Western blot (WB) analysis showed that while the T, P, and P + T treatments inhibited HER phosphorylation and downstream PI3K/AKT and ERK signaling, the inhibition was suboptimal and did not reach statistical significance (Fig. 3a). N-containing regimens, on the other hand, were highly effective in blocking the HER pathway, as seen by inhibited pHER2, pEGFR, pHER3, pAKT, pERK, and pS6 protein levels (Supplementary Fig. 5). Further, though there was some inter-tumor variation, overall we observed reduced total HER2 levels in the N and N + T-treated tumors, which is in line with previous reports of N-mediated degradation of the HER2 receptor^23^. Furthermore, the substantial tumor regression observed with N-containing regimens was further supported by our observation of great inhibition in the levels of the anti-apoptotic marker Survivin and marginal increase in the levels of the pro-apoptotic marker cleaved PARP (c-PARP), compared to T and P, either alone or in combination (Fig. 3a). Likewise, the mitotic activity, as assessed by levels of phospho histone 3 (pH3) was significantly inhibited upon treatment with N, both alone and in combination with T (Supplementary Fig. 5). Consistent with the WB results, immunohistochemistry (IHC) analysis showed substantial inhibition in the pHER2, pAKT, and pERK levels in tumors treated with the N-containing but not antibody-containing regimens (Fig. 3b, c). In addition, tumor cell proliferation, measured by %Ki67, was significantly inhibited only in the groups treated with N-containing regimens. Further, the combination of N + T significantly outperformed P + T in inhibiting tumor cell proliferation in this HER2-amplified xenograft model (Fig. 3b, c).

### Neratinib-containing but not antibody-containing treatments markedly inhibit HER signaling pathway and tumor cell proliferation in BCM-3963 PDX model

**Fig. 4:**
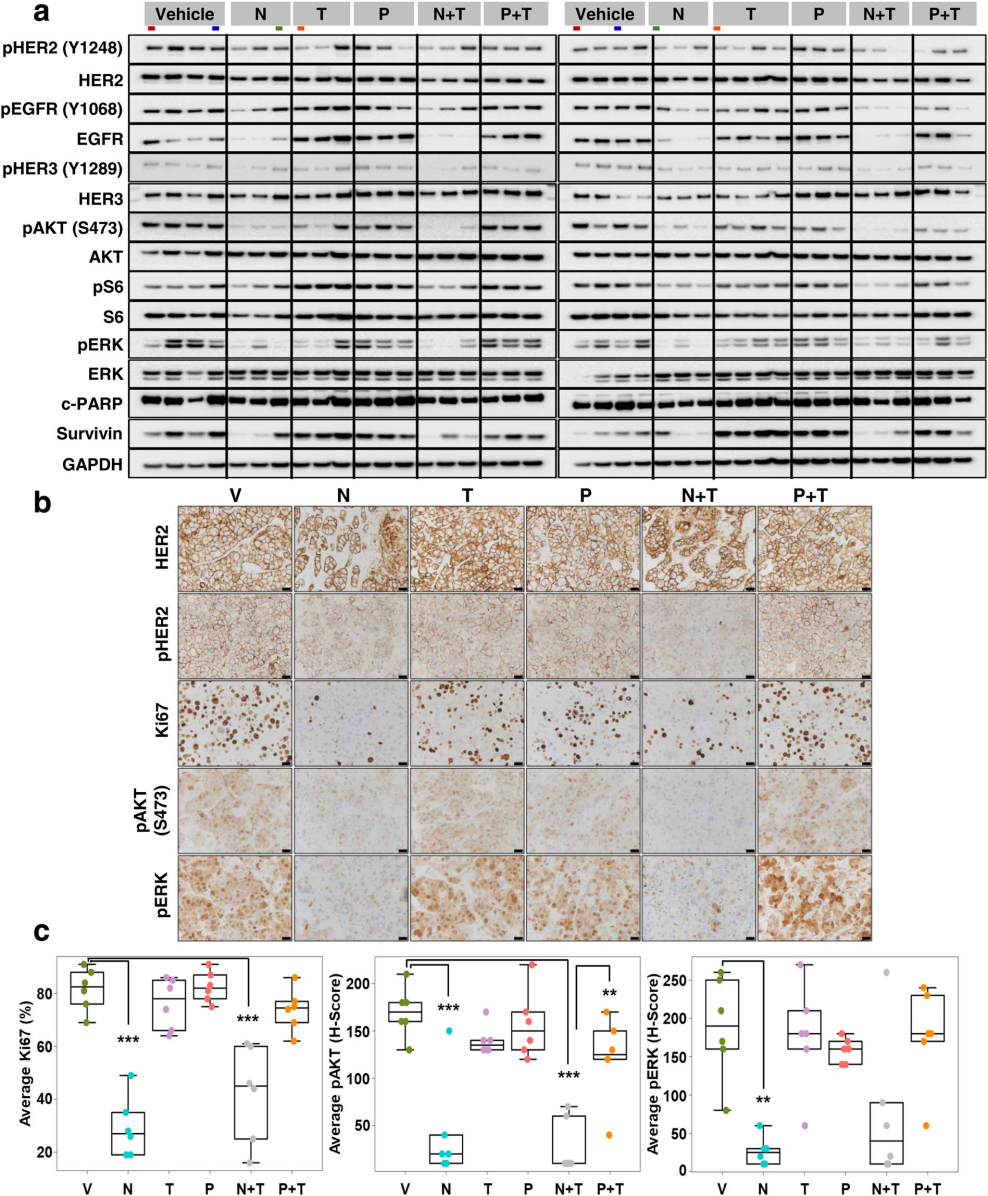
Neratinib-containing regimens suppress cell proliferation and HER2 signaling in short-term treated BCM-3963 patient-derived xenografts. **a** Western blot analyses of alterations in level and activation of proteins along the HER signaling axis. Each of the six short-term treatment arms had six tumors, which were run in two separate gels with each gel containing three tumors per arm. Colored bars below the treatment arms in gray boxes denote duplicate samples loaded as comparison control between left and right blots. **b** Representative HER2, pHER2, Ki67, pAKT, and pP44/42 ERK immunohistochemical staining. **c** Box plots showing average Ki67 (%), pAKT (*H-*Score), and pERK (*H*-Score) protein levels by immunohistochemistry. Box plots indicate median and interquartile range (IQR). The lower and upper hinges correspond to the first (25th percentile) and third (75th percentile) quartiles, respectively. The whiskers extend to about 1.5 × IQR from the hinge, and data points beyond the whiskers are suspected outliers. V vehicle, N neratinib, T trastuzumab, P pertuzumab. IHC images in **b** are of ×40 magnification, Scale bar: 50 μm. **P < 0.01, ***P < 0.001.

BCM-3963 tumor-bearing mice (*n* = 6) were randomized to short-term treatment with either vehicle, N, T, P, N + T, or P + T, and the harvested tumors were subjected to molecular analysis. WB analysis showed that the N-containing regimens effectively inhibited the HER signaling pathway, as seen by substantially inhibited pHER2, pEGFR, pHER3, pAKT, pERK, and pS6 levels (Fig. 4a and Supplementary Fig. 6). In contrast, T and P, both alone and in combination, failed to effectively inhibit the HER signaling pathway. Interestingly, like the BT474 model, we observed slightly downregulated total HER2 levels and greatly suppressed total EGFR levels in the N and N + T treated tumors, which could be due to the activity of N against various HER dimers. As with the BT474 model, the substantial tumor regression observed with N-containing regimens was further supported by greatly reduced levels of the anti-apoptotic protein Survivin in the PDX model as well (Fig. 4a). Likewise, the mitotic activity, as assessed by the levels of phospho histone 3 (pH3) was significantly inhibited upon treatment with N or N + T, but not with T and P, either alone or in combination (Supplementary Fig. 6). IHC analysis showed marked inhibition in pHER2, pAKT, and pERK levels in tumors treated with N-based but not antibody-containing regimens (Fig. 4b, c). Further, compared to the tumors treated with antibody-containing regimens, tumor cell proliferation was significantly suppressed in tumors treated with N-containing regimens (Fig. 4b, c). This observation is in line with the superior anti-tumor efficacy of N-based regimens over antibody-containing regimens. Additionally, the tumors treated with N and N + T showed lower tumor cellularity and areas of smeared HER2 staining suggesting massive cell death, and were more fibrotic, indicative of the clearing and replacement of dead tumor cells with fibrous tissue. Although less apparent compared to the BCM-3963 tumors, lower tumor cellularity was also observed in the N and N+T treated BT474 xenografts.

To exclude the possibility that the failure of antibody-containing regimens to inhibit HER2 signaling and tumor cell proliferation is likely due to shorter treatment duration, and to ensure buildup of sufficient and stable plasma drug levels, we conducted a second short-term study to test whether an extended pre-treatment with the antibodies will promote their anti-tumor efficacy. Mice bearing BCM-3963 tumors (*n* = 6) were randomized to either vehicle, N, N + T, or P + T arms. For combination treatments, the antibodies were administered twice over a span of 4 days, and N treatment began the day after second antibody dose and continued every day for the next 4.5 days, with a third antibody dose on day 4 of N treatment. IHC analysis of the short-term treated tumors showed that, despite the longer treatment duration, P + T failed to inhibit the Ki67 levels. On the other hand, N and N + T treatments significantly inhibited the HER signaling and Ki67 levels, thereby confirming the superiority of N-containing treatments over anti-HER2 antibodies in this HER2-amplified PDX model (Supplementary Fig. 7).

Finally, we sought to determine the efficacy of N in comparison with L, a less potent inhibitor of HER1 than HER2 that has demonstrated efficacy, especially in combination with T, in the neoadjuvant clinical setting^9^. The BCM-3963 tumors treated with N and L, either alone or in combination with T, were subjected to molecular analyses. Interestingly, WB analysis showed that N was superior to L, both as a single agent and in combination with T, in blocking the HER receptors, as seen by markedly inhibited pEGFR, pHER4, and phospho and total HER2 and HER3 levels (Supplementary Fig. 8). The extent of inhibition in the phosphorylated levels of downstream signaling molecules such as AKT, ERK, and S6, however, was largely similar between the tumors treated with lapatinib- or neratinib-containing regimens. Further, N, both alone and in combination with T, greatly reduced the levels of the anti-apoptotic protein Survivin (Fig. 4a). Likewise, IHC analysis revealed that N offered a better inhibition of Ki67 levels compared to L (Supplementary Fig. 9). When combined with T, both N and L achieved significant inhibition in tumor cell proliferation (Supplementary Fig. 9).

## Discussion

In addition to HER2, signaling from other HER members^19^ is important in activating the HER pathway, and we previously reported that combining a HER1 inhibitor (e.g., gefitinib) with the HER2 mAbs T and P further enhanced the anti-tumor efficacy and accelerated tumor eradication^7^. Thus, with newer agents such as neratinib (N), which more potently inhibit HER1 (EGFR) and the various HER dimers, compared to other currently approved small-molecule agents, including the dual HER1/2 TKI lapatinib (L) and the selective HER2 TKI tucatinib, we are better positioned in our pursuit of treating the truly HER2-addicted tumors, especially with HER2-targeted therapy alone, without chemotherapy. HER2-addicted tumors are associated with high levels of HER2 and display complete functional dependence on HER2 and lack genetic and/or functional aberrations in key components of downstream signaling pathways, such as the PI3K/AKT pathway (e.g., *PIK3CA* mutations)^18,24^. A very recent preclinical study in fact showed that regression of HER2-positive breast cancer PDX models with the potent H1047 *PIK3CA* mutations could only be achieved with the addition of everolimus to neratinib, but not with neratinib alone^25,26^. In addition, the anti-HER2-sensitivity of HER2-enriched tumors is comparatively higher than other subtypes and studies have shown that the HER2-enriched subtype is more likely to benefit from chemotherapy-sparing HER2-targeted therapy alone^16^. Indeed, both the BT474 and BCM-3963 xenograft models used in this study are highly HER2-amplified and HER2-dependent with a HER2-enriched intrinsic subtype^20,22^. While the BCM-3963 model harbors wild-type *PIK3CA*, the BT474 model harbors a functionally weak *PIK3CA* mutation (K111N), which does not jeopardize its functional dependence on HER2^6,7^.

We recently reported through neoadjuvant trials that L + T, with endocrine therapy if ER+, but without chemotherapy, yields meaningful response in HER2+ breast cancer^15,16,27^. The efficacy of N in the early setting, especially in combination with T, and how it compares to P + T or L + T, particularly in the absence of chemotherapy, has not been carefully explored. In this study, using both ER+ and ER− HER2+ breast cancer xenograft models of different genetic background, we demonstrate the great potency of N, either alone or in combination with T, two targeted agents with complementary mechanisms of action, in achieving tumor regression and eradication compared to T and P, either alone or together. Clinically, the FB-7 neoadjuvant trial comparing neratinib, trastuzumab, or the combination, with chemotherapy, in patients with locally advanced HER2+ breast cancer, showed a numerically greater pCR rate with neratinib+trastuzumab compared to single agents^28,29^. Neratinib+trastuzumab yielded a significantly higher pCR rate in the hormone receptor (HR)-negative tumors, compared to the HR+ tumors (*P* = 0.001), which, however, did not receive endocrine therapy.

Our biomarker studies demonstrate the overall efficacy of N, either alone or in combination with T, in achieving a comprehensive blockade of the HER pathway and its downstream signaling, and in more effectively inhibiting cell proliferation, compared to P + T. Specifically, the inhibitory effect of T on pERK levels, either alone or in combination with P, varied depending on the xenograft model, ranging from substantial inhibition in the T-sensitive BT474 model to minimal inhibition in the T-refractory BCM-3963 model. Importantly, compared to T and P, either alone or together, N + T accelerated tumor regression in both models and further inhibited the pERK levels, including in the highly T-sensitive BT474 model. Effective inhibition of additional downstream HER signaling, especially AKT that is driven predominantly by the HER heterodimers beyond the HER2 homodimers, calls for the use of a HER2 TKI, particularly to inhibit the ligand-dependent HER3 heterodimers^30,31^. Indeed, in both xenograft models, we observed that N and N + T markedly suppressed the levels of pHER receptors, owing to N being a pan-HER TKI and a more potent inhibitor of the HER signaling, as opposed to the mAbs. Further, a substantial inhibition in the total EGFR, HER2, and HER3 levels was observed with neratinib, both alone and even more so in combination with trastuzumab. Our findings are in line with recent preclinical reports showing downregulation of total HER receptor levels in HER2-positive cell models treated with neratinib^32,33^. In contrast, with lapatinib alone, the phospho and total HER3 levels were either marginally increased or remained unaffected, potentially suggesting the feedback upregulation of HER3 levels following lapatinib treatment^8,34^. The addition of trastuzumab to lapatinib, however, induced a modest inhibition in the total HER3 levels, possibly due to the ability of trastuzumab to disrupt, at least partly, the HER2-HER3 dimers^31^. Overall, our data further underscore the notion that adding a second HER2-targeted agent with a different mechanism of action, especially a TKI that can inhibit all the HER dimers, can more effectively inhibit the downstream signaling of the entire HER receptor layer.

While the combination of T and P has greatly improved outcomes, resistance does occur, as with the BCM-3963 model, which in an immunocompromised setting is de novo refractory to T and P, both as a single-agent and in combination. Molecular analyses showed that the two antibodies did not have a marked inhibitory effect on HER and its downstream signaling. This could presumably be, at least partly, due to the failure of T and P to block EGFR, an important activator of the HER pathway, as well as, at least partly due to signaling stemming from ligand-activated HER receptor complexes involving HER3 and/or HER4^35^. Consistent with the clinical behavior of the original patient tumor^22^, the combination of L + T was effective in regressing the BCM-3963 tumors. Importantly, in this model, we showed the functional superiority of N, either alone or together with T, in terms of both anti-tumor efficacy and in suppressing the HER pathway, over the clinically effective combination of P + T and L + T, which holds great clinical implications, especially in the neoadjuvant setting to spare chemotherapy. Our findings are in line with previous reports showing the efficacy of N in overcoming intrinsic and acquired resistance to T^36^.

In addition to the neoadjuvant setting, this treatment combination may also be highly relevant and extended to the metastatic setting. An international phase I/II study reported that neratinib in combination with trastuzumab was well tolerated and had promising anti-tumor activity^37^. In the phase III NALA trial, the risk of disease progression or death was reduced by 24% with neratinib+capecitabine vs lapatinib+capecitabine (*P* = 0.006)^38^. Further, the NEfERT-T study showed reduction in the frequency of CNS recurrences and improvement in the time to occurrence of CNS events in the neratinib-paclitaxel group compared to trastuzumab-paclitaxel group^39^, which further emphasizes the need to include a potent small-molecule TKI (e.g., neratinib) that can cross the BBB to access the overt and micro-metastasis in the CNS, in both early and advanced settings. In addition, a recent case study showed that 3 HER2-amplified metastatic colorectal cancers with acquired resistance to P + T still derived benefit from adding a TKI, L to T^40^. Thus, TKIs, especially the more potent second-generation pan-HER agents in combination with T, given their different mechanisms of action, could potentially overcome resistance to dual anti-HER2 antibody therapy by more comprehensively inhibiting the HER receptor layer and overcoming resistance due to survival signaling from the uninhibited HER receptor(s), p95 HER2 that may be effectively targeted by L but not by T^41,42^, and excess ligand-mediated activation of HER1/3/4 in various HER dimers^43^, which is also relevant in the metastatic niches.

One of the challenges with agents that effectively suppress EGFR, including N, is adverse events (AEs) like diarrhea and rash due to inhibition of EGFR expressed in the gastrointestinal mucosa and skin^44–48^. Diarrhea, particularly in the first 1–2 months, is the main tolerability concern with neratinib. In a chemotherapy-free neoadjuvant setting, without the added toxicity of chemotherapy, the AEs associated with N may be more clinically manageable with appropriate prophylactic regimens, a notion that warrants clinical testing. Further, interestingly, in both our HER2-amplified preclinical models with different genetic backgrounds, tumor eradication was achieved even with 20 mg/kg N, which is half the recommended human equivalent dose of 240 mg/kg^38^. Further, this dose was well tolerated in both models, which eliminated the reasoning of tumor regression because of systemic toxicity. Although extrapolating this notion directly to humans is difficult, our data suggest the possibility that a highly HER2-amplified tumor could be effectively treated with a lower dose, especially when used in combination with T, similar to our previous report that when used in combination with T, half the dose of L is as effective as full dose in eradicating the BT474 tumors^6^. Lower doses of single-agent neratinib have not been widely studied and formal exposure–response relationships have not been described. Several phase 2 studies have demonstrated clinical benefit and good tolerability when lower doses of neratinib (160 mg/kg) are combined with other HER2-directed therapies such as T-DM1^49,50^. In the phase 2 CONTROL study evaluating antidiarrheal strategies for neratinib in early-stage HER2+ breast cancer, using the weekly-dose escalation regimen with neratinib during the first weeks of therapy was associated with a low incidence of Grade 3 diarrhea, but was also associated with fewer treatment discontinuations and dose holds^51^ (Ruiz-Borrego et al., SABCS 2020, Abstract PS13-20). Thus, adoption of neratinib dose escalation may significantly improve the tolerability and long-term adherence to neratinib, a strategy that warrants further testing in the clinical setting.

A potential limitation of this study is the use of immunocompromised models to investigate the anti-tumor efficacy of mAbs, which leverage the host immune system to facilitate effector cell-mediated ADCC to kill cancer cells. While the athymic nude mice have some NK cell activity, the SCID/Beige mice are devoid of NK cells^22^, but bear monocytes and macrophages, and are therefore capable of enabling partial ADCC mediated by these effectors^22,52,53^. The lack of a fully functional immune system in our study models may have impacted the efficacy of certain agents, particularly the mAbs, to some extent. However, this model offered crucial insights into the anti-tumor activity of the HER2-targeted agents at the molecular/signaling level, which is equally important in evaluating their treatment efficacy. Further, as has been shown in a multitude of previous preclinical studies using such mouse models, including ours, results from these studies in general prove to be of great clinical relevance for the intended patient population.

Together, in this study, we have established the therapeutic potential of neratinib, either alone or together with trastuzumab in eradicating HER2-amplified breast cancer. Further, our data call attention to the rationale of combining anti-HER2 agents such as neratinib and trastuzumab with complementary mechanisms of action to more effectively treat HER2-amplified breast cancers and circumvent resistance. Importantly, this treatment combination may be effective in the neoadjuvant chemotherapy-free setting to treat highly HER2-addicted tumors to spare the toxicity from unwarranted chemotherapy, without imperiling patient outcomes. Our findings have immediate and important clinical implications and strongly support clinical studies to test the efficacy of this combination in the early-stage clinical setting, without chemotherapy for HER2+ breast cancer.

## Methods

### Cell lines and reagents

BT474/AZ cells were maintained in Dulbecco’s modified Eagle’s medium (DMEM) supplemented with 10% heat-inactivated fetal bovine serum (FBS) and 1% penicillin–streptomycin–glutamine^7,8^. The cell line was authenticated at the MD Anderson Characterized Cell Line Core Facility within 6 months of performing the experiments and was tested to be mycoplasma-free by MycoAlert™ Mycoplasma Detection Kit (Lonza). Trastuzumab and pertuzumab were purchased from Mckesson Specialty Health, and formulated in sterile water or sterile 1× PBS, respectively^6,7^. Neratinib (provided by Puma Biotechnology Inc.) was formulated in 0.5% methylcellulose, 0.4% Tween-80. Lapatinib (LC Laboratories) was diluted in a 1% Tween-80 solution in autoclaved water.

### Xenograft studies

All animal studies were conducted in accordance with and approved by the Institutional Animal Care and Use Committee (IACUC) of Baylor College of Medicine.

#### BT474/AZ cell xenografts

5 × 10^6^ BT474/AZ (ER+/HER2+) cells suspended in 1:4 matrigel were injected subcutaneously into 5–6-week-old female athymic mice (Envigo, USA) supplemented with 0.36 mg 60-day-release estrogen pellets implanted subcutaneously^7,54^. When the tumors reached 250–350 mm^3^, mice were randomized to short-term (*n* = 5–7 mice/arm; treated for 3.5 days) or long-term treatment (*n* = 9–14 mice/arm) with vehicle (0.5% methylcellulose, 0.4% Tween-80) ± estrogen deprivation (ED) by removal of estrogen pellets, and neratinib (20 mg/kg, once daily oral gavage, 5 days/week), trastuzumab (10 mg/kg, IP twice a week), pertuzumab (6 mg/kg, IP twice in the first week followed by 6 mg/kg once a week), neratinib+trastuzumab, or pertuzumab+trastuzumab, all under ED.

#### BCM-3963 patient-derived xenografts (PDX)

Fragments of freshly dissected BCM-3963 (ER−/HER2+) PDX tumors were orthotopically transplanted into cleared mammary fat pads of 3-4 week-old female SCID/Beige mice (Envigo, USA)^22^. Mice were palpated weekly and tumor growth measured using calipers. When tumors reached 250–350 mm^3^, mice were randomized to short-term (*n* = 5–7 mice/arm; treated for 4.5 days) or long-term treatment (*n* = 13–19 mice/arm) with vehicle, neratinib, trastuzumab, pertuzumab, neratinib+trastuzumab, or pertuzumab+trastuzumab. In some short-term experiments, mice were also randomized to lapatinib (100 mg/kg, once daily oral gavage, 5 days/week) or lapatinib+trastuzumab. For evaluation of the therapeutic efficacy of lapatinib, the BCM-3963 tumor-bearing mice were randomized to either vehicle (1% Tween-80 solution in autoclaved water), lapatinib (100 mg/kg, once daily oral gavage, 5 days/week), trastuzumab (10 mg/kg, IP twice a week), or the combination of lapatinib and trastuzumab.

For both models, tumors were measured twice per week using a caliper and tumor volumes (mm^3^) were calculated by the formula (width)^2^ × length/2 using the measures of the two largest tumor diameters^7,8,55,56^. Mice were sacrificed and tumors were harvested 4 h after the last treatment for analysis of associated biomarkers (for short-term experiments), or when they reached 1000 mm^3^ or at completion of the experiment, except when tumor eradication was achieved (for long-term studies). Tumor tissues were preserved in liquid nitrogen or formalin-fixed and paraffin embedded (FFPE) for later analyses. To determine whether the tumor cells were completely eradicated in treatment groups that achieved complete response, treatment was stopped when no palpable tumor was detected for ~120 days on-treatment and mice were maintained under continued ED. Two weeks after treatment cessation, some mice were further randomized to E2 supplementation. Mice in both E2 and ED groups were monitored for additional ~60 days for tumor recurrence.

### Immunoblotting assays

Proteins were extracted from BT474/AZ and BCM-3963 tumor tissues using RIPA lysis buffer, supplemented with protease and phosphatase inhibitor cocktails and PhosStop (Roche). Twenty micrograms of each sample were separated by electrophoresis on NuPAGE Novex 4–12% Bis-Tris Gels (Invitrogen) and transferred onto nitrocellulose membranes using the iBlot® 2 Dry Blotting System (Invitrogen)^21^. The blots were visualized by chemiluminescence on a ChemiDoc™ Touch Imaging System, and analyzed using the Image Lab Software Version 5.2.1 (Bio-Rad). Each western blot image was generated from one gel. For each gel, duplicate sets of samples were run for parallel blotting of the phospho- and total-specific antibodies of the same protein marker. Blots were blocked with 5% milk in PBS with 0.1% Tween-20 (PBST) and incubated with respective primary antibodies diluted 1:1000 in 5% BSA in PBST (Supplementary Table 1). Blots were washed in PBST and then incubated with HRP-linked secondary antibody (Cell Signaling) diluted 1:2000 in 5% milk in PBST. Blots were incubated in primary antibodies overnight at 4 °C and in secondary antibody for 1 h at room temperature. All western blot images were quantified using the Bio-rad Image Lab™ software. Each background-adjusted protein band volume was normalized to the respective GAPDH value. The GAPDH-adjusted band volume of each treatment replicate was then normalized to the average GAPDH-adjusted band volume of the vehicle group in the respective experiment. All blots are derived from the same experiment and were processed in parallel. Images of uncropped blots are presented in the Supplementary file.

### Immunohistochemistry

BT474/AZ and BCM-3963 tumor tissues were fixed overnight in 4% neutral-buffered formalin, washed with 70% ethanol, and processed for paraffin embedding. After deparaffinization of the FFPE sections, endogenous peroxidase was blocked by 3% H_2_O_2_, and endogenous avidin and biotin were blocked by the AB blocking kit (Vector, Burlingame, CA)^7^. Immunohistochemistry (IHC) was then performed on 4-µm sections using Ki67, pan-cytokeratin AE1/AE3, HER2, pHER2 Y1221/1222, pAKT S473, and pERK antibodies (Supplementary Table 1). Tumors were scored by the percentage of positive cells for Ki67 and pH3, and by *H*-score for pAKT and pERK staining^7,57^.

### Statistical analysis

Tumor growth curves were generated using the mean tumor volume for each time point and error bars represent the standard error of mean (SEM). Study endpoints included time to tumor regression (TTR; defined as days to the first observation of tumor size halving relative to the size at randomization) and progression (TTP; defined as days to the first observation of tumor size doubling relative to the size at randomization), and the incidence and time to complete response (CR and TCR, respectively, TCR; defined as days to the first observation of complete tumor disappearance). Time to tumor doubling and halving is linearly interpolated from the data to estimate the exact time point when the tumor volume reached twice or half the randomization volume. CR rates were calculated based on total number of animals in each long-term treatment group. If the tumor size had not reached an event threshold by the last observation, it was considered as censored at the last time point. TTP, TTR, and TCR survival curves were estimated using the Kaplan–Meier method, summarized by median and 95% CI (loglog transform) and compared by the generalized Wilcoxon test and corrected for multiple comparisons. For the BT474 model global test was performed first, followed by unadjusted pairwise comparisons of interest, while for the BCM-3963 model only two groups were compared. Differences in Ki67%, and pAKT and pERK *H*-scores from the IHC analysis, and WB quantification were summarized as box plots or as scatter bar plots with mean ± SEM and results were compared by one-way or two-way ANOVA with Bonferroni multiple comparisons correction using GraphPad Prism V6.05.

### Reporting summary

Further information on research design is available in the Nature Research Reporting Summary linked to this article.

## Supplementary information

Supplementary Information

Reporting Summary

## Data Availability

The data generated and analyzed during this study are described in the following data record: 10.6084/m9.figshare.14453193^58^. The tumor growth and body weight data, western blot quantification data and biomarker immunohistochemistry quantification data are openly available in the figshare repository via the following 10.6084/m9.figshare.13284146^59^. The uncropped western blots are available in the Supplementary Materials.
