# Supplementary material for: Neratinib plus trastuzumab is superior to pertuzumab plus trastuzumab in HER2-positive breast cancer xenograft models
Source: NPJ Breast Cancer. 2021 May 27;7:63. doi: 10.1038/s41523-021-00274-0 (PMC8159999; doi:10.1038/s41523-021-00274-0)

**Supplementary Information:**

**Supplementary Figure 1**. Schematic of the study design to evaluate the anti-tumor efficacy of single or dual anti-HER2 treatment regimens.

**Supplementary Figure 2**. (**a**) Changes in the volume of individual BT474-AZ xenografts over time upon various treatments. In each drug treatment group, treatments were stopped after ~120 days of no palpable tumor on treatment. After 2 weeks, some mice were randomized to E2 supplementation while the rest were maintained under continued ED, and all mice were monitored for ~60 days for recurrence. (**b**) Average changes in body weight over time of BT474/AZ xenograft-bearing mice. Mean±SEM, E2, estrogen; ED, estrogen deprivation; Veh, vehicle; N, neratinib; T, trastuzumab; P, pertuzumab.

**Supplementary Figure 3**. (**a**) Growth curves showing changes in the tumor volume of individual BCM-3963 PDX over time upon various anti-HER2 treatments. In the neratinib and neratinib+trastuzumab treatment groups, drug treatments were stopped after ~120 days of no palpable tumor on-treatment and all mice were monitored for ~60 days for recurrence. (**b**) Average changes in body weight over time of BCM-3963 PDX-bearing mice. Mean±SEM, Veh, vehicle; N, neratinib; T, trastuzumab; P, pertuzumab.

**Supplementary Figure 4**. **Trastuzumab enhances the anti-tumor efficacy of lapatinib in BCM-3963 patient-derived xenograft model**. Growth curves showing changes in the tumor volume of individual BCM-3963 patient-derived xenografts treated with (**a**) vehicle, (**b**) trastuzumab, (**c**) lapatinib, or (**d**) lapatinib+trastuzumab. **The average tumor volumes of vehicle and lapatinib+trastuzumab treatment arms were published previously (*Zhang et al, Cancer Res. 2013 Aug 1;73(15):4885-97*).

**Supplementary Figure 5**. **Neratinib containing regimens significantly inhibit HER2 signaling and mitotic activity in short-term treated BT474 xenograft tumors**. (**a**) Grouped dot plots of target proteins quantified from western blot analysis showing alterations in the levels and activation of signaling proteins along the HER signaling axis. P-values are in comparison to the vehicle treated group. Results are presented as mean ± SEM. (**b**) Representative phospho histone 3 (pH3) immunohistochemical staining images (*top panel*) and box plots showing average pH3 (%) protein levels by immunohistochemistry (*bottom panel*). Veh, Vehicle; N, neratinib; T, trastuzumab; P, pertuzumab. One-way ANOVA with Bonferroni's correction. * P<0.05, ** P<0.01, ***P<0.001, **** P<0.0001.

**Supplementary Figure 6**. **Neratinib containing regimens significantly inhibit HER2 signaling and mitotic activity in short-term treated BCM-3963 patient-derived xenograft tumors**. (**a**) Grouped dot plots of target proteins quantified from western blot analysis showing alterations in the levels and activation of signaling proteins along the HER signaling axis. P-values are in comparison to the vehicle treated group. Results are presented as mean ± SEM. (**b**) Representative phospho histone 3 (pH3) immunohistochemical staining images (*top panel*) and box plots showing average pH3 (%) protein levels by immunohistochemistry (*bottom panel*). Veh, Vehicle; N, neratinib; T, trastuzumab; P, pertuzumab. One-way ANOVA with Bonferroni's correction. * P<0.05, ** P<0.01, ***P<0.001, **** P<0.0001.

**Supplementary Figure 7**. **Neratinib but not trastuzumab or pertuzumab suppress tumor cell proliferation and HER2 signaling in short-term treated BCM-3963 PDX tumors.** (**a**)

Representative Ki67, pAKT, pP44/42 ERK, and HER2 IHC staining, and (**b**) Box plot showing the average Ki67 (%) protein levels by immunohistochemistry. N, neratinib; T, trastuzumab; P, pertuzumab. Box plots indicate median and interquartile range (IQR). The lower and upper hinges correspond to the first ($25^{th}$ percentile) and third ($75^{th}$ percentile) quartiles, respectively. The whiskers extend to about 1.5 × IQR from the hinge, and data points beyond the whiskers are suspected outliers. IHC images in panel (a) are of 40X magnification, Scale bar: 50µm. **** P<0.0001.

**Supplementary Figure 8**. **Neratinib- and lapatinib-containing regimens significantly inhibit HER signaling in short-term treated BCM-3963 PDX tumors**. (**a**) Western blot analysis, and (**b**) Grouped dot plots representing quantification of target proteins from panel (a) showing alterations in the level and activation of signaling proteins along the HER signaling axis. In panel (a), colored bars below the treatment arms in grey boxes denote duplicate samples loaded as comparison control between the left and right blots. Results are presented as mean ± SEM. Veh, Vehicle; N, neratinib; L, lapatinib; T, trastuzumab; P, pertuzumab. One-way ANOVA with Bonferroni's correction. * P<0.05, ** P<0.01, ***P<0.001. P-values are in comparison to the vehicle treated group.

**Supplementary Figure 9**. **Neratinib- and lapatinib-containing regimens suppress tumor cell proliferation and HER2 signaling in short-term treated BCM-3963 PDX tumors**. (**a**) Representative Ki67, pHER2 (Y1221/1222), and HER2 IHC staining, and (**b**) Box plot showing the average Ki67 (%) protein levels by immunohistochemistry. Box plots indicate median and interquartile range (IQR). The lower and upper hinges correspond to the first ($25^{th}$ percentile) and third ($75^{th}$ percentile) quartiles, respectively. The whiskers extend to about 1.5 × IQR from the hinge, and data points beyond the whiskers are suspected outliers. IHC images in panel (a) are of 40X magnification, Scale bar: 50µm. N, neratinib; L, lapatinib; T, trastuzumab; P, pertuzumab. * P<0.05, ** P<0.01, **** P<0.0001.

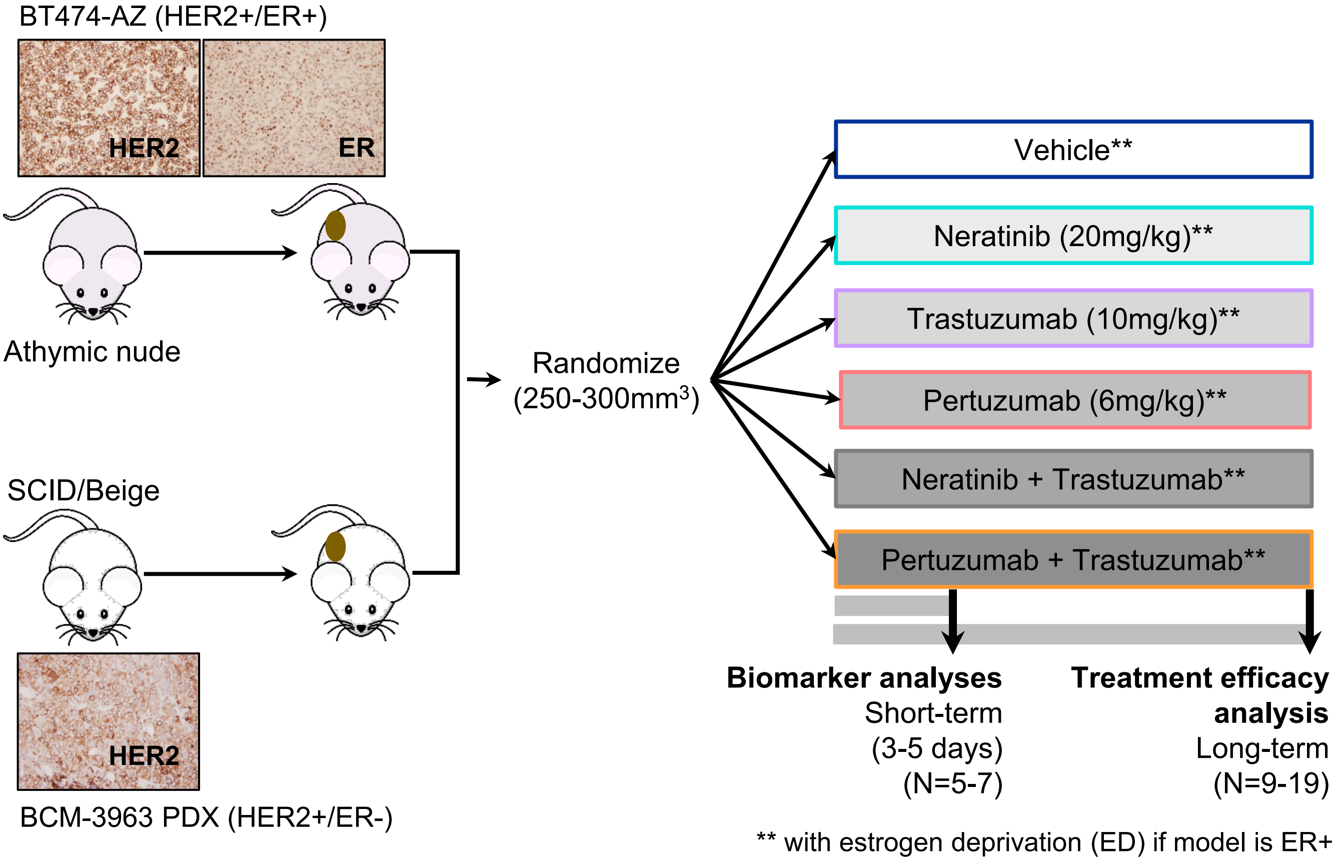


**Supplementary Figure 1**

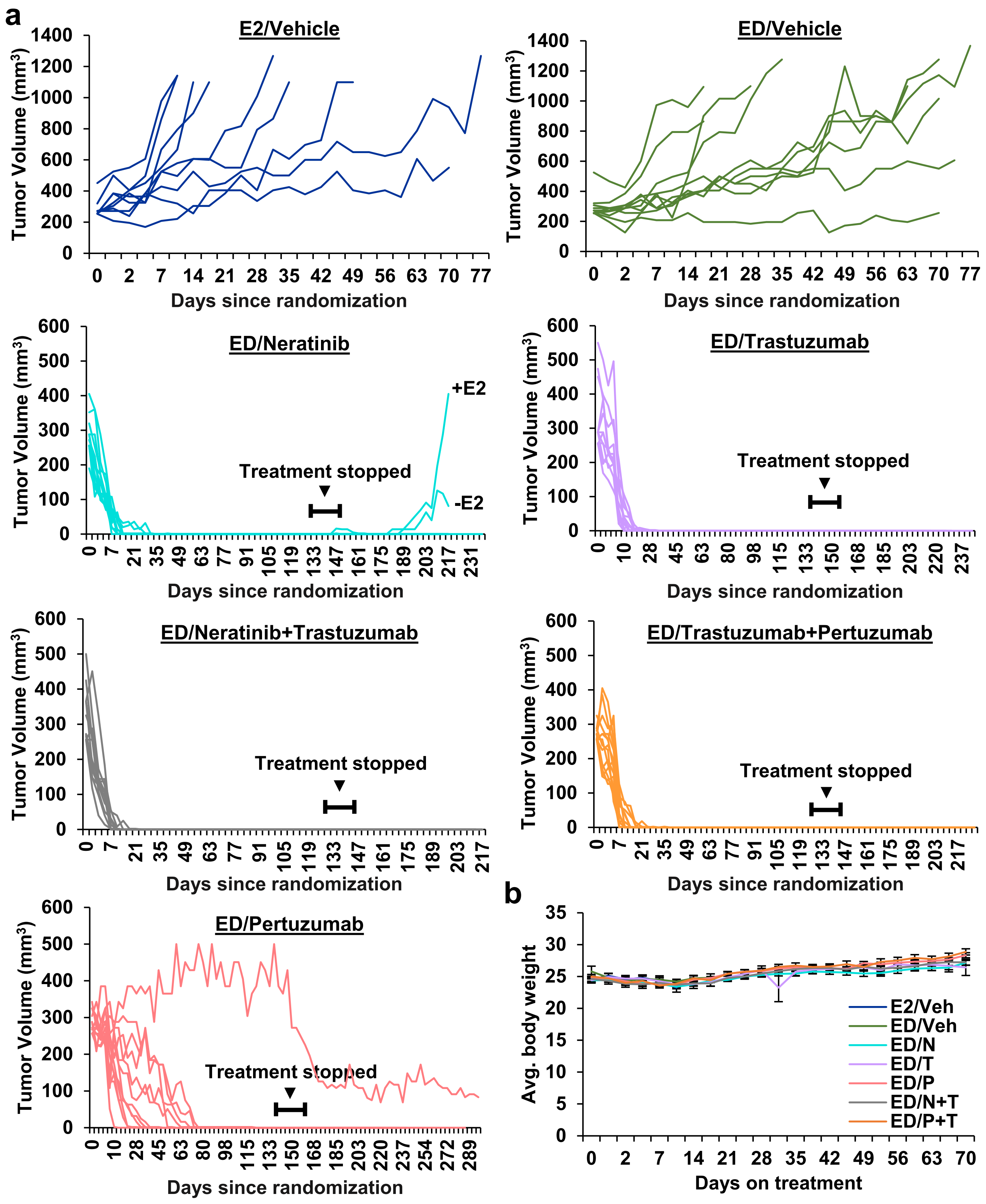


Supplementary Figure 2

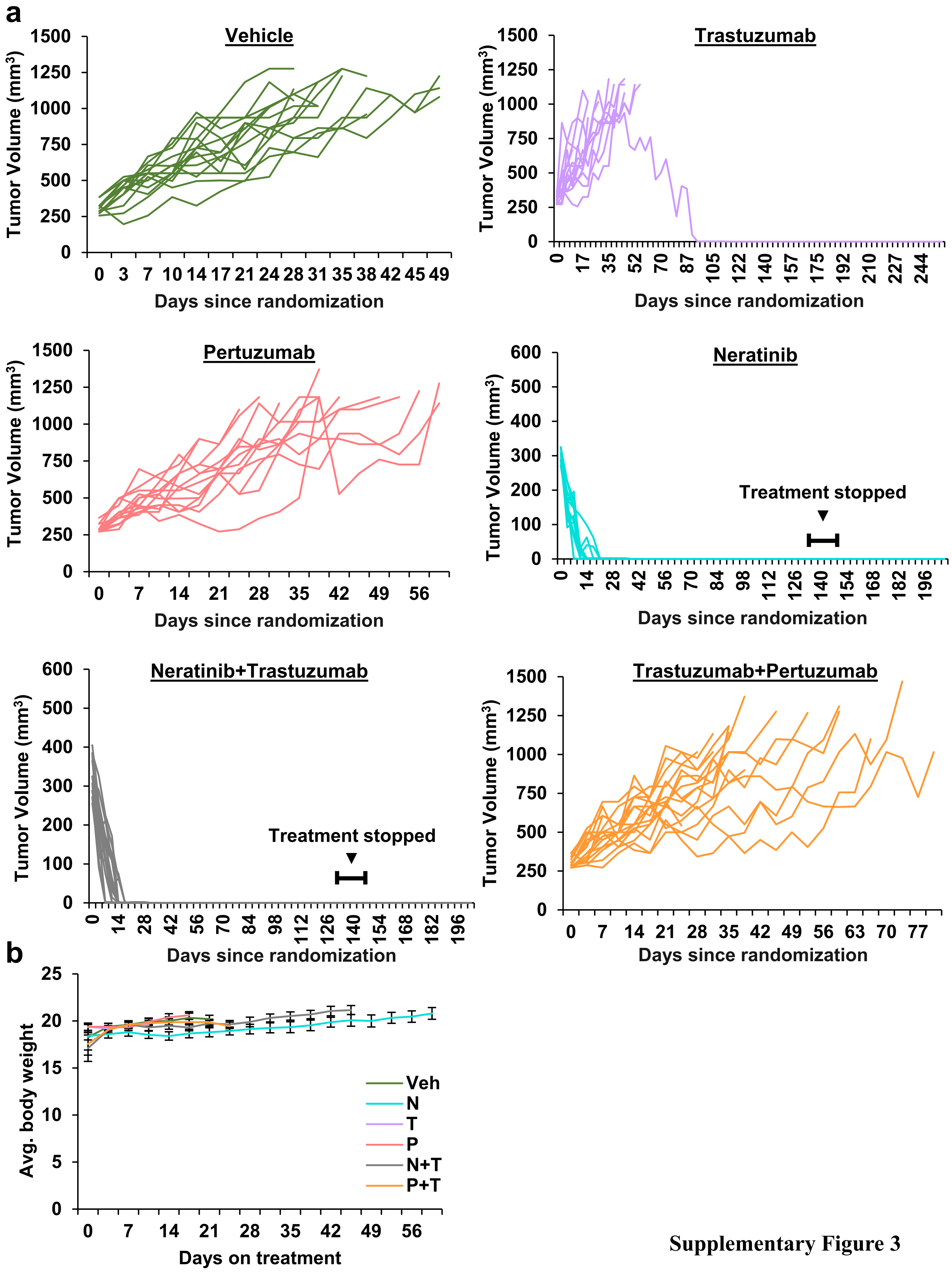


Supplementary Figure 3

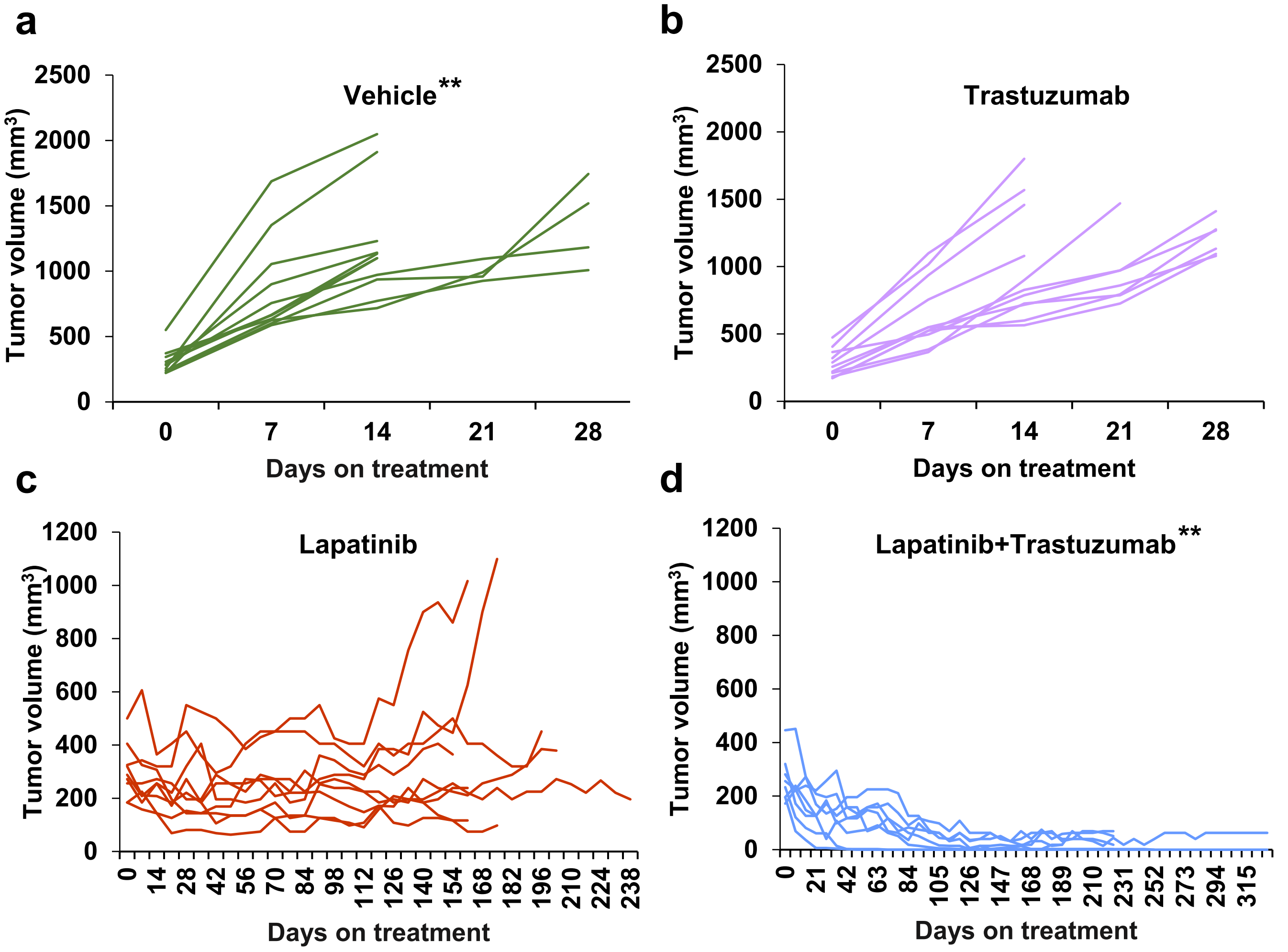


**Supplementary Figure 4**

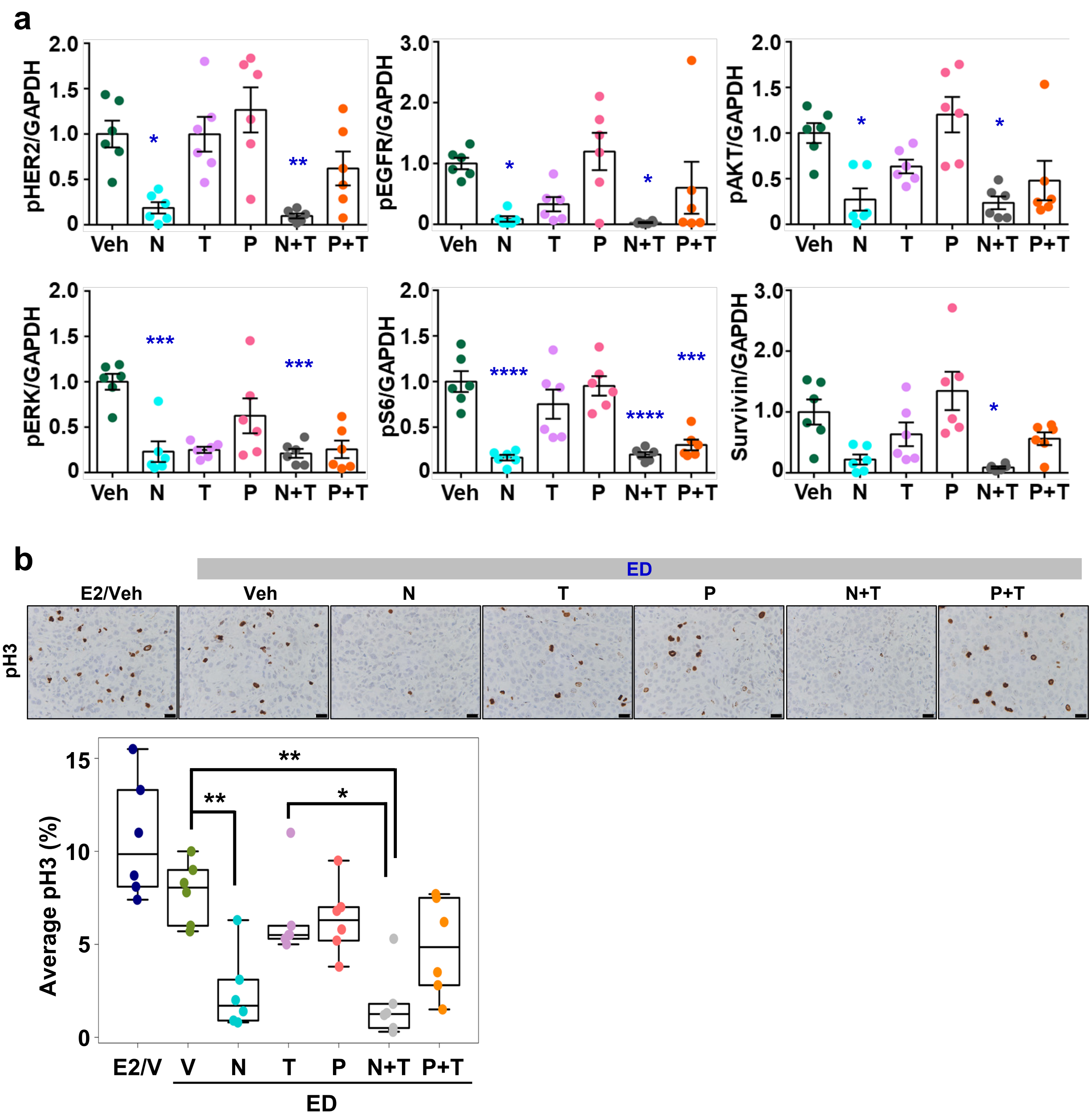


Supplementary Figure 5

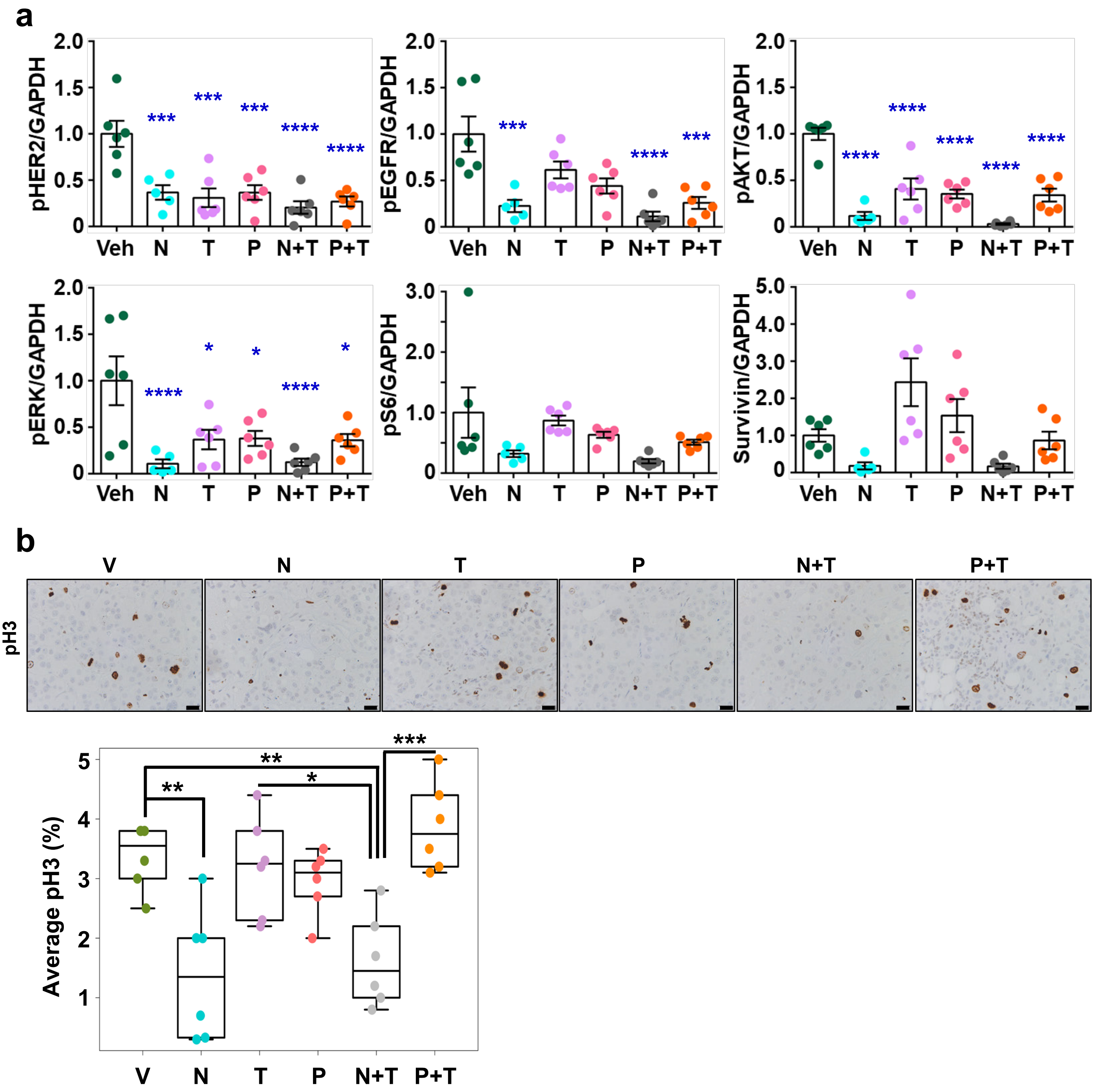


Supplementary Figure 6

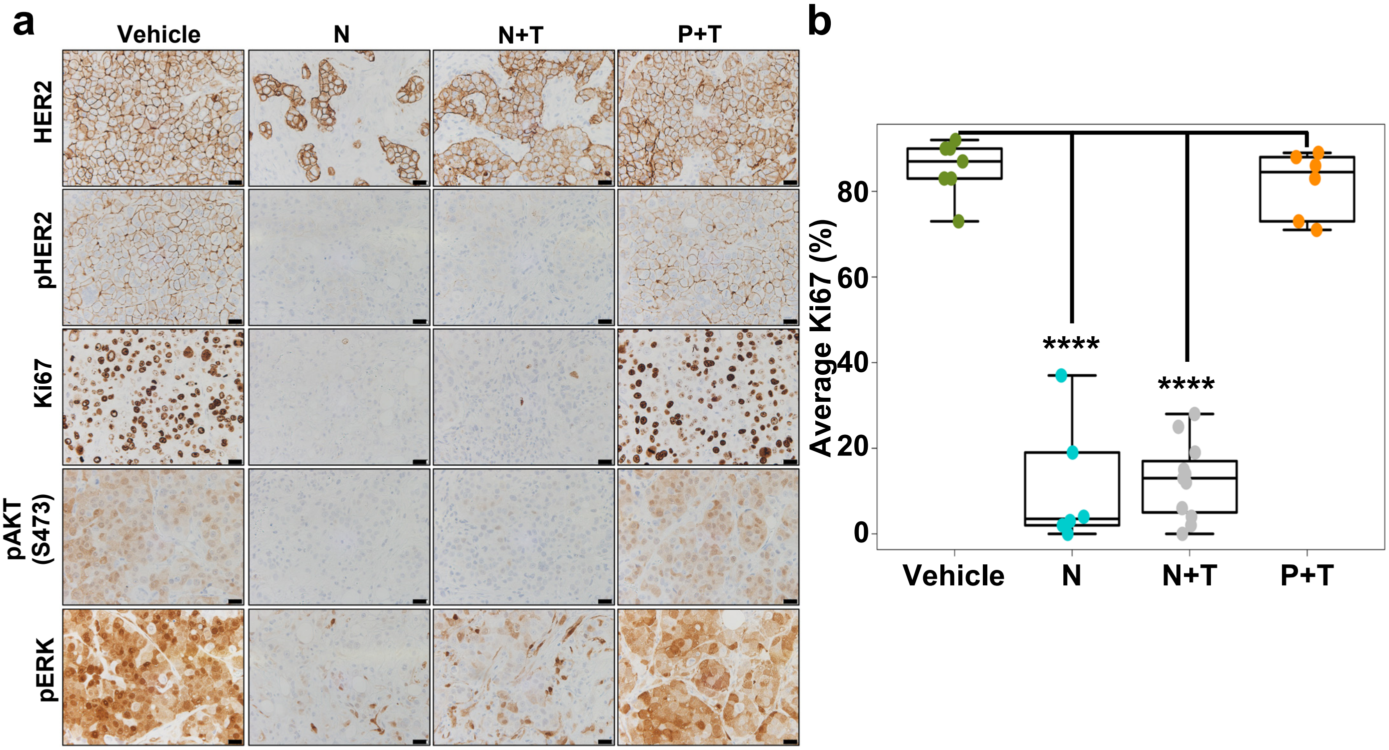


Supplementary Figure 7

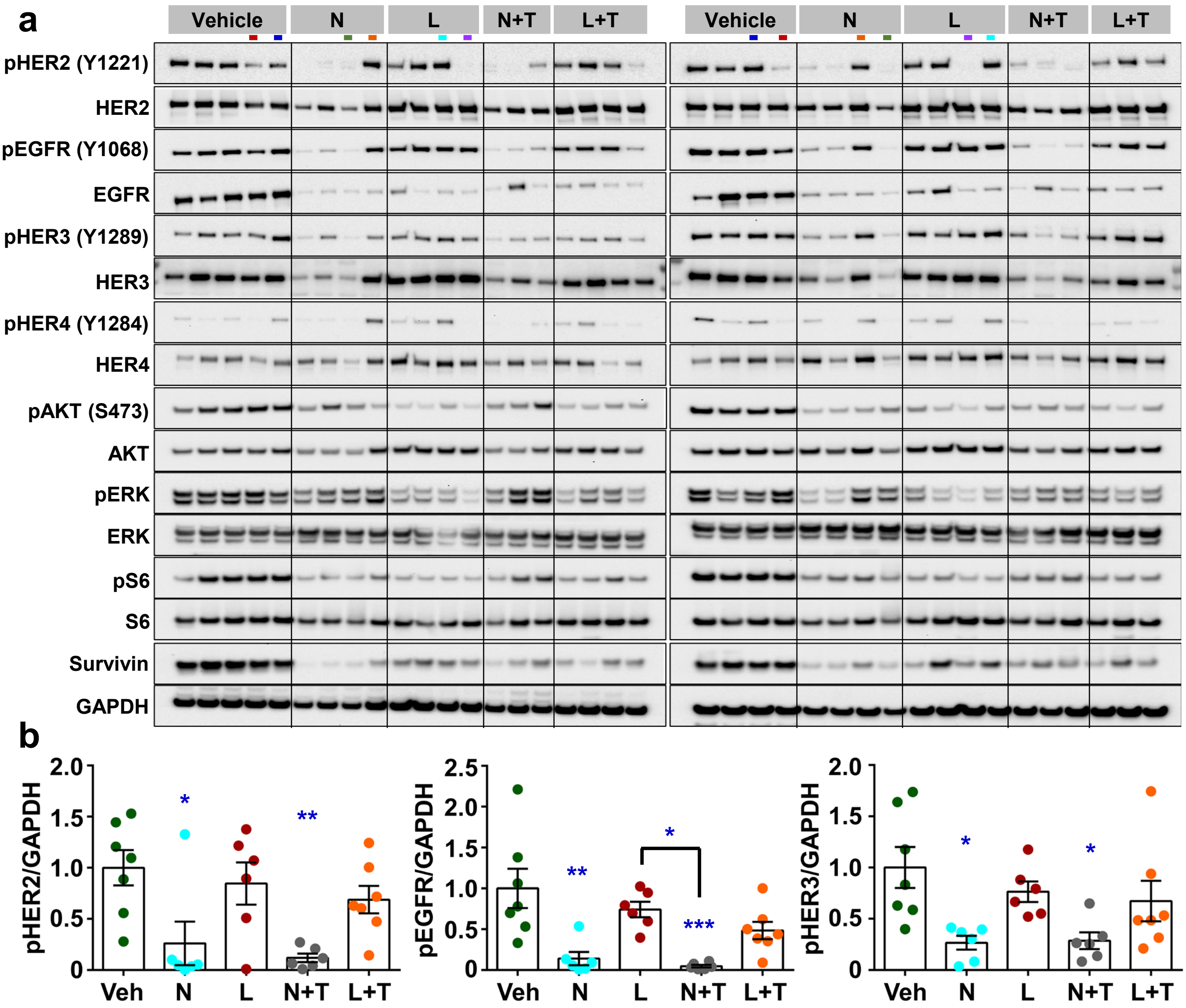


Supplementary Figure 8

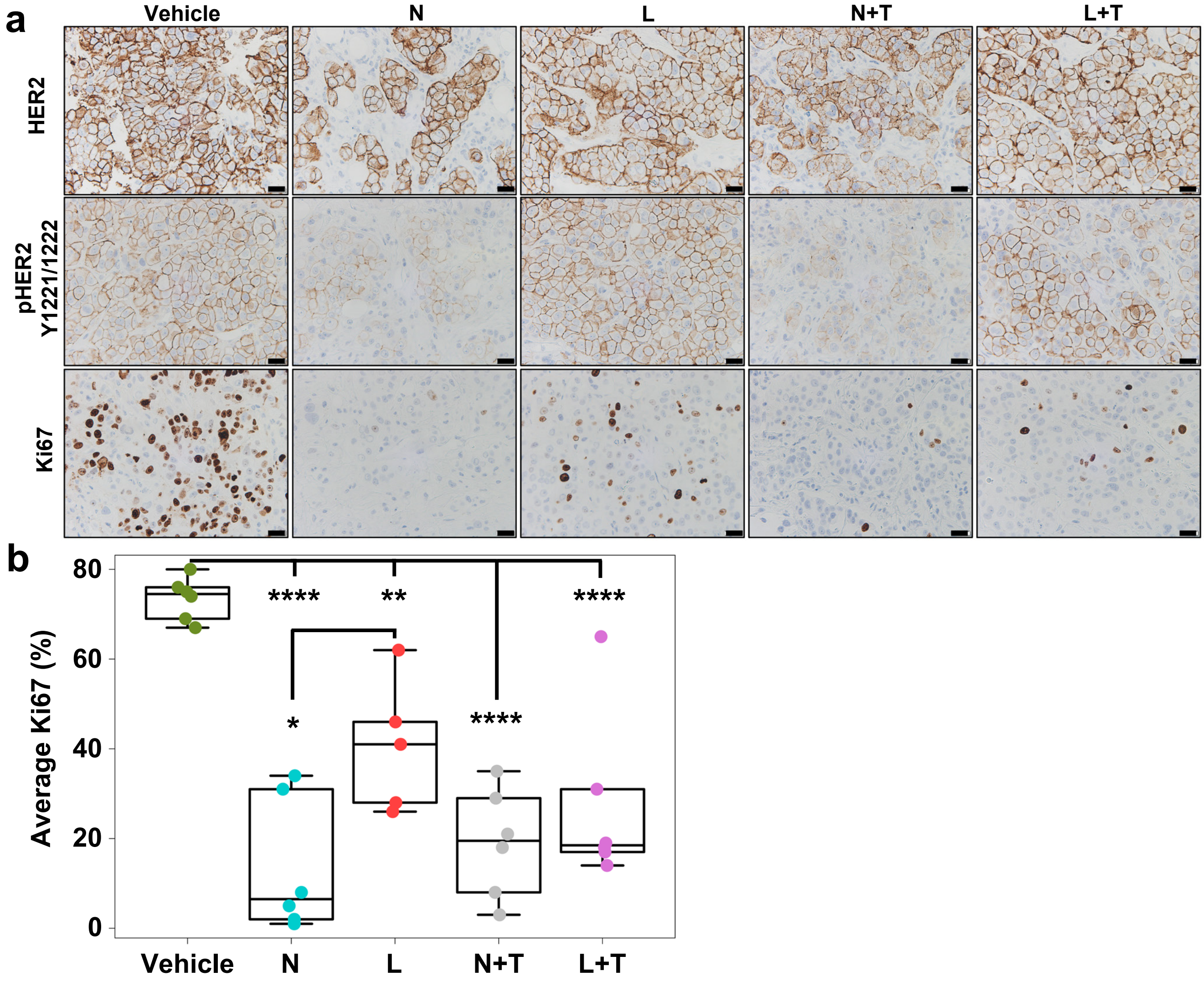


Supplementary Figure 9

**Supplementary Table 1.** Details of primary antibodies used for immunoblotting and immunohistochemistry assays

| Primary Antibody | Supplier | Catalog No. | Dilution used |
|---|---|---|---|
| **Western Blot** | | | |
| pHER2_Y1221/1222 | Cell Signaling Technology | 2243S | 1:1000 |
| pHER2_Y1248 | Cell Signaling Technology | 2247S | 1:1000 |
| HER2 | Calbiochem | OP15 | 1:1000 |
| pEGFR_Y1068 | Cell Signaling Technology | 2231S | 1:1000 |
| EGFR | Cell Signaling Technology | 2232S | 1:1000 |
| pHER3_Y1289 | Cell Signaling Technology | 4791S | 1:1000 |
| HER3 | Millipore | 05-390 | 1:1000 |
| pHER4_Y1284 | Cell Signaling Technology | 4757T | 1:1000 |
| HER4 | Cell Signaling Technology | 4795S | 1:1000 |
| pERK1/2_T202/204 | Cell Signaling Technology | 9101S | 1:1000 |
| ERK1/2 | Cell Signaling Technology | 9102S | 1:1000 |
| pAKT_S473 | Cell Signaling Technology | 4060S | 1:1000 |
| AKT | Cell Signaling Technology | 9272S | 1:1000 |
| pS6_S235/236 | Cell Signaling Technology | 2211S | 1:1000 |
| S6 | Santa Cruz Biotechnology | SC-74576 | 1:1000 |
| Survivin | Cell Signaling Technology | 2808S | 1:1000 |
| c-PARP | Cell Signaling Technology | 9541S | 1:1000 |
| GAPDH | Santa Cruz Biotechnology | SC- 365062 | 1:1000 |
| **Immunohistochemistry** | | | |
| Ki67 | Dako | M7240 | 1:200 |
| Pan-cytokeratin AE1/AE3 | Dako | M3515 | 1:1000 |
| HER2 | Thermo Fisher | RM-9103-S-A | 1:100 |
| pHER2_Y1221/1222 | Cell Signaling Technology | 2243L | 1:50 |
| pAKT_S473 | Cell Signaling Technology | 3787L | 1:50 |
| pERK1/2_T202/204 | Cell Signaling Technology | 9101L | 1:80 |
| pH3 | Upstate | 09-797 | 1:250 |

# Uncropped western blot images of Figure 3a

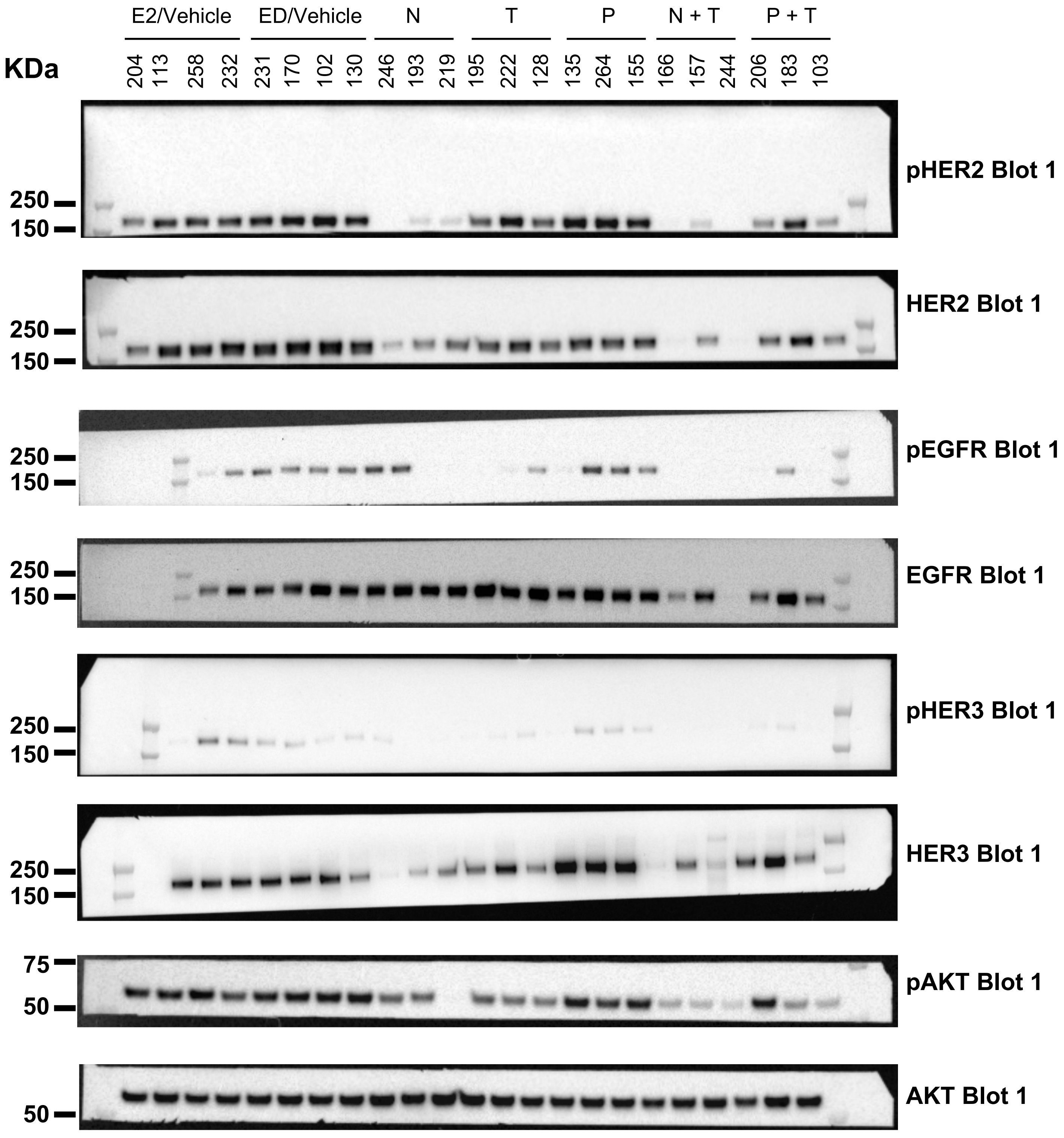

# Uncropped western blot images of Figure 3a

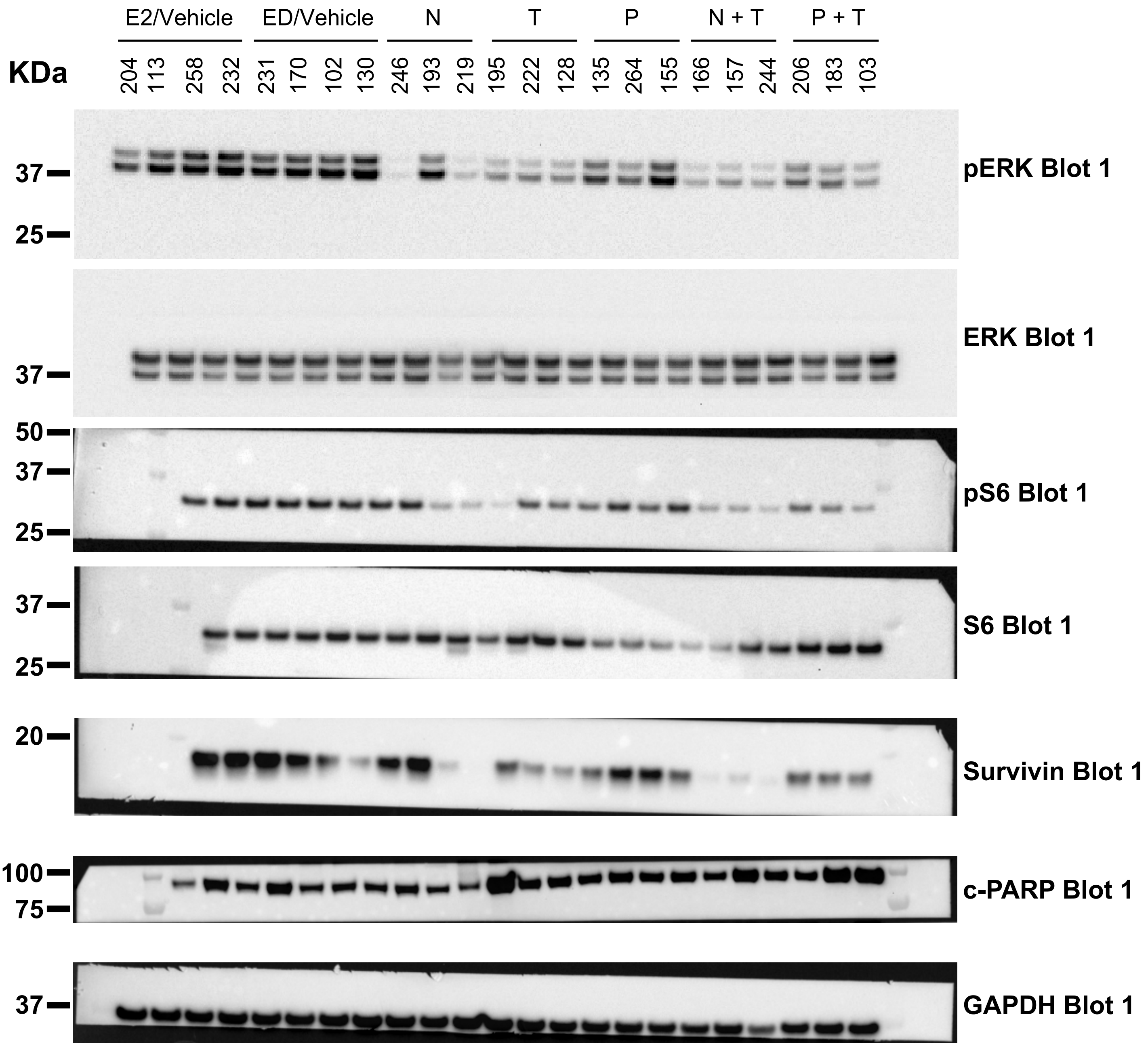

## Uncropped western blot images of Figure 3a

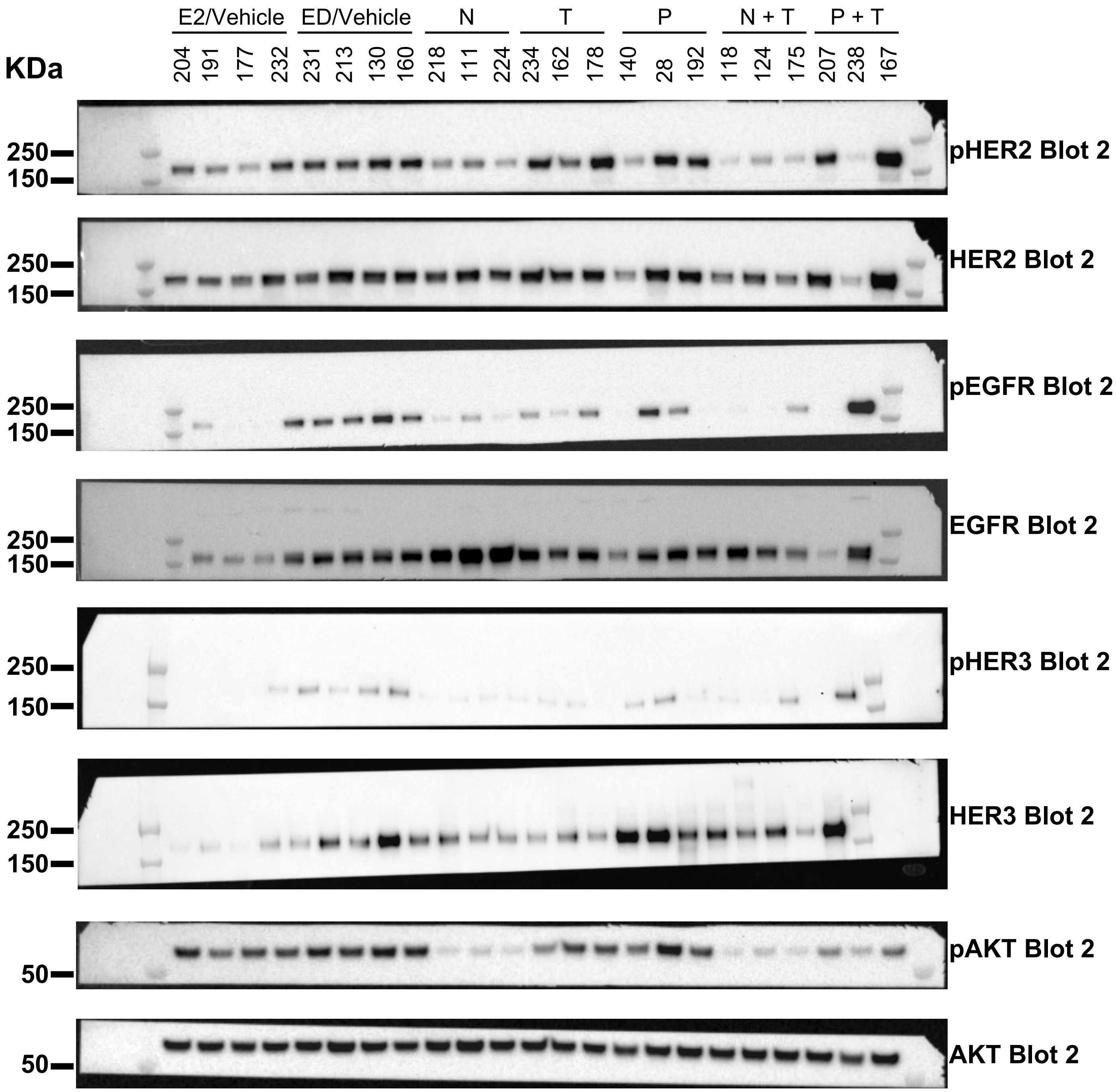

## Uncropped western blot images of Figure 3a

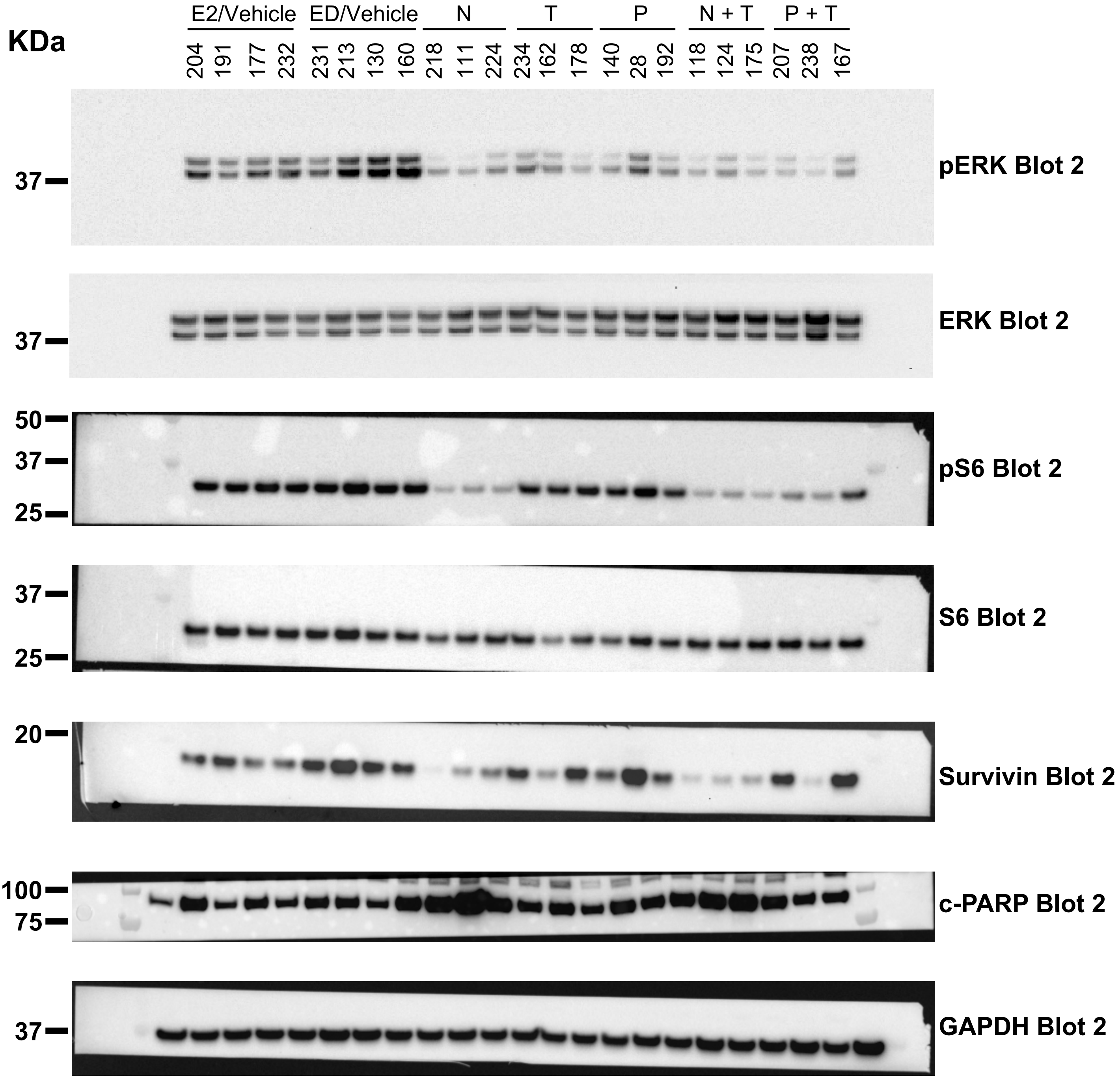

## Uncropped western blot images of Figure 4a

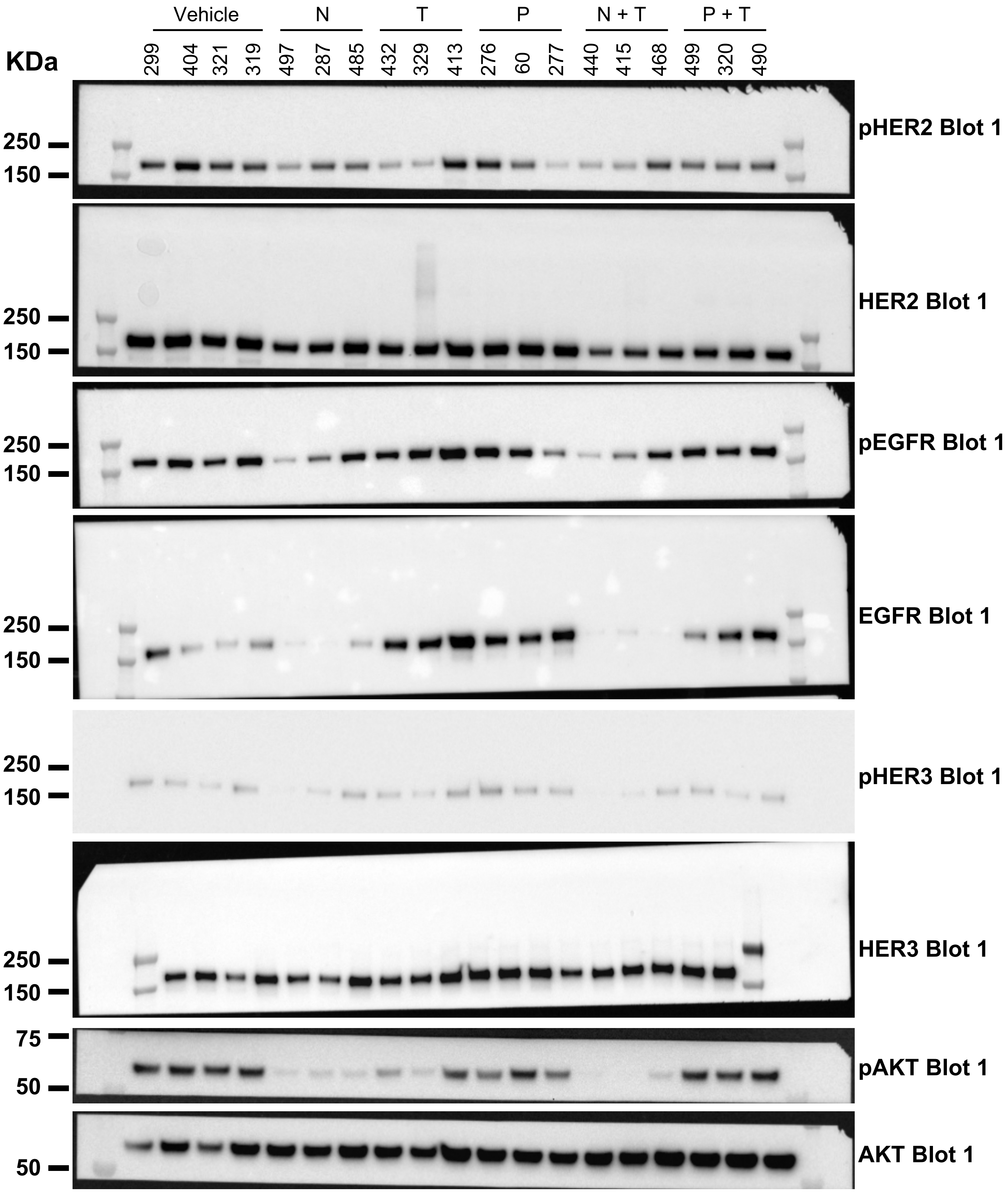

## Uncropped western blot images of Figure 4a

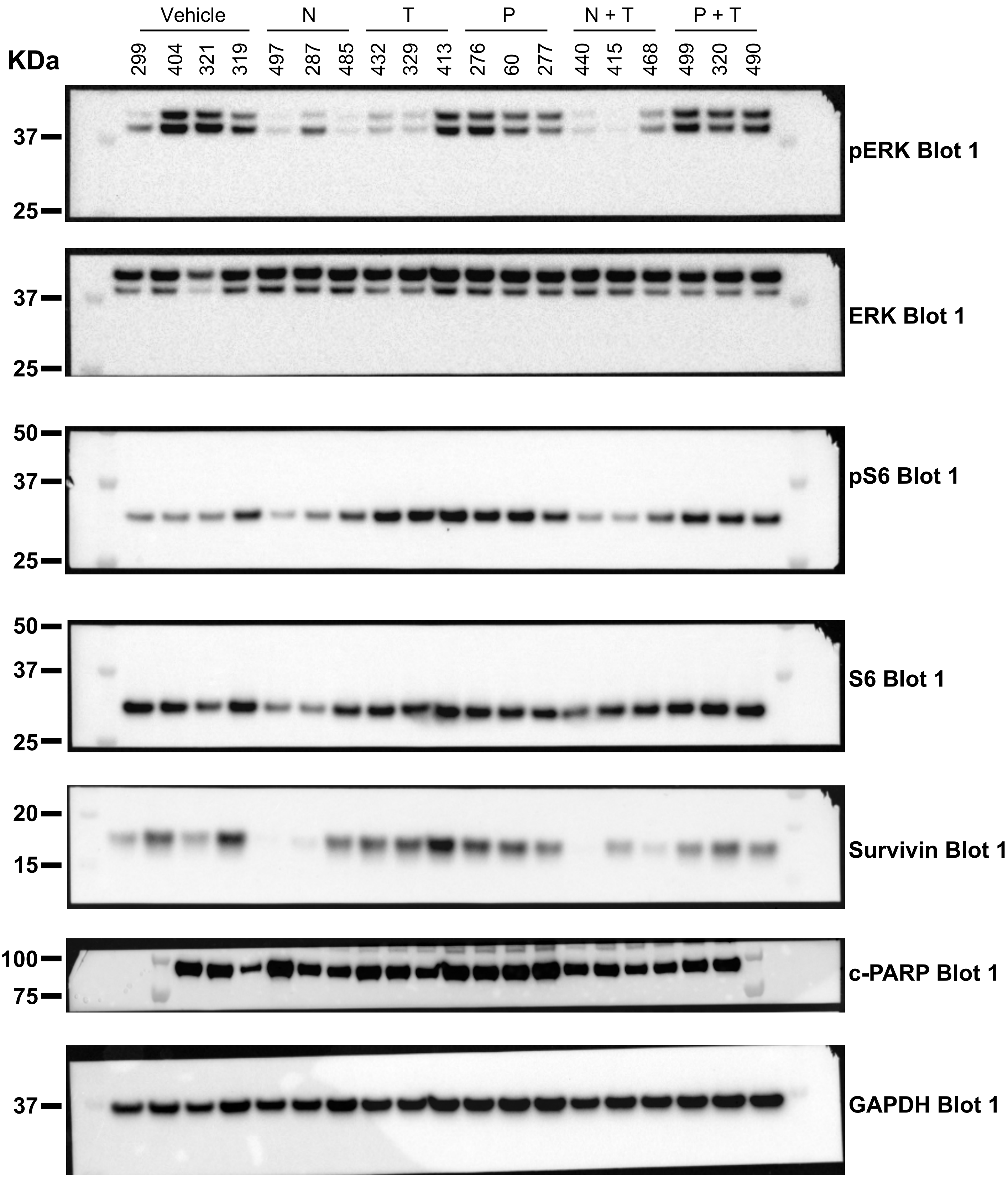

## Uncropped western blot images of Figure 4a

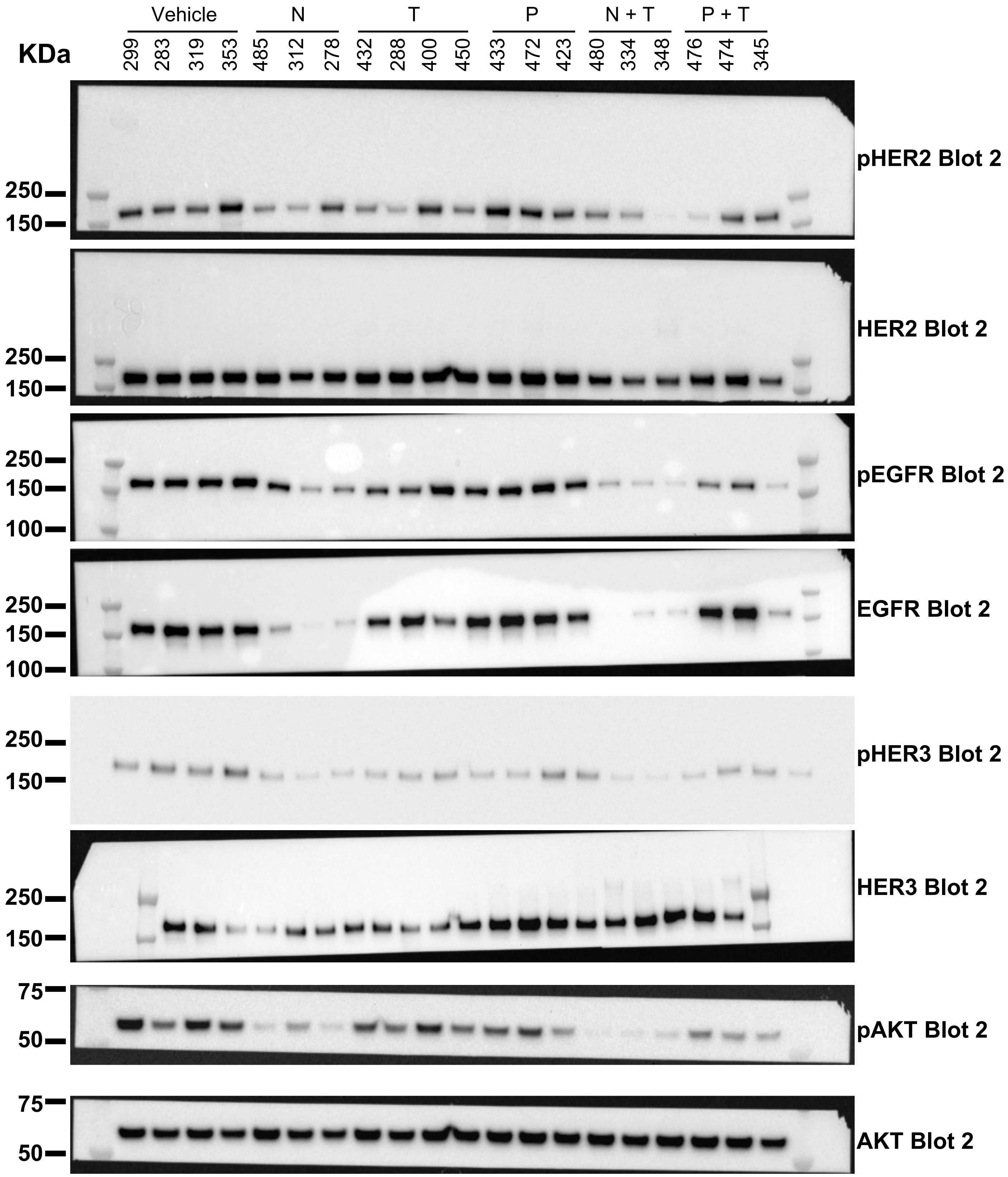

## Uncropped western blot images of Figure 4a

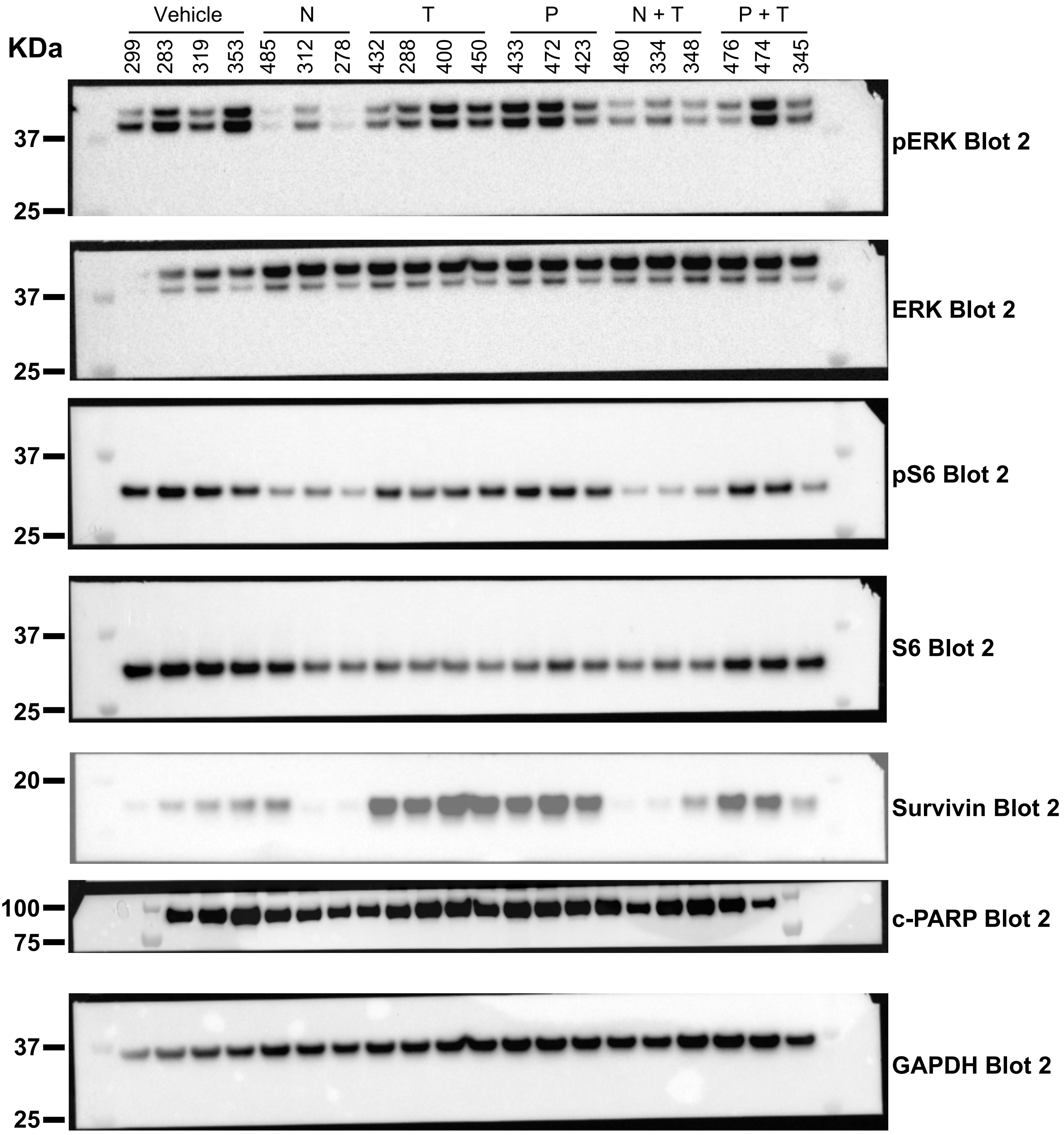

# Uncropped western blot images of Supplementary figure 8a

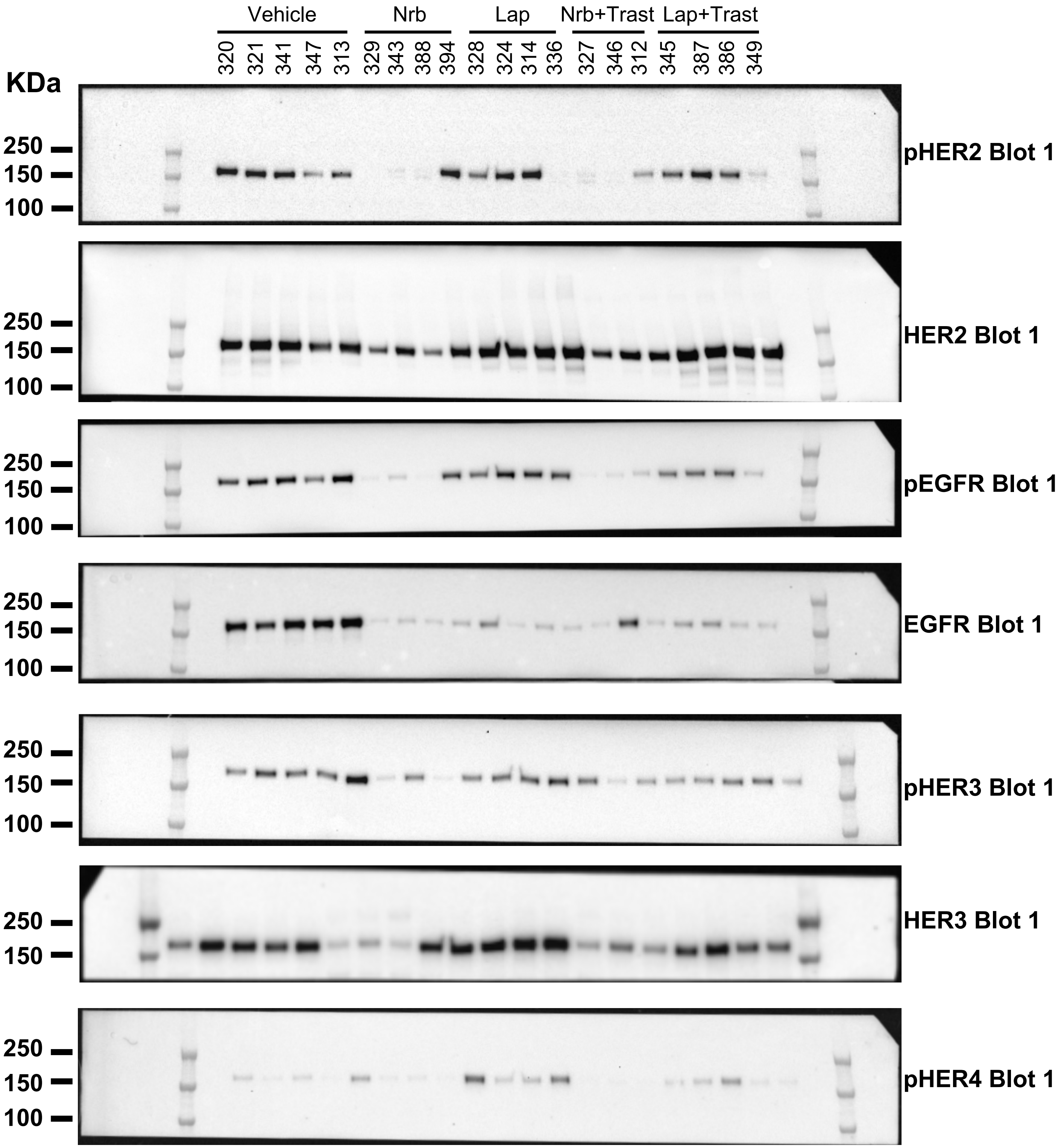

# Uncropped western blot images of Supplementary figure 8a

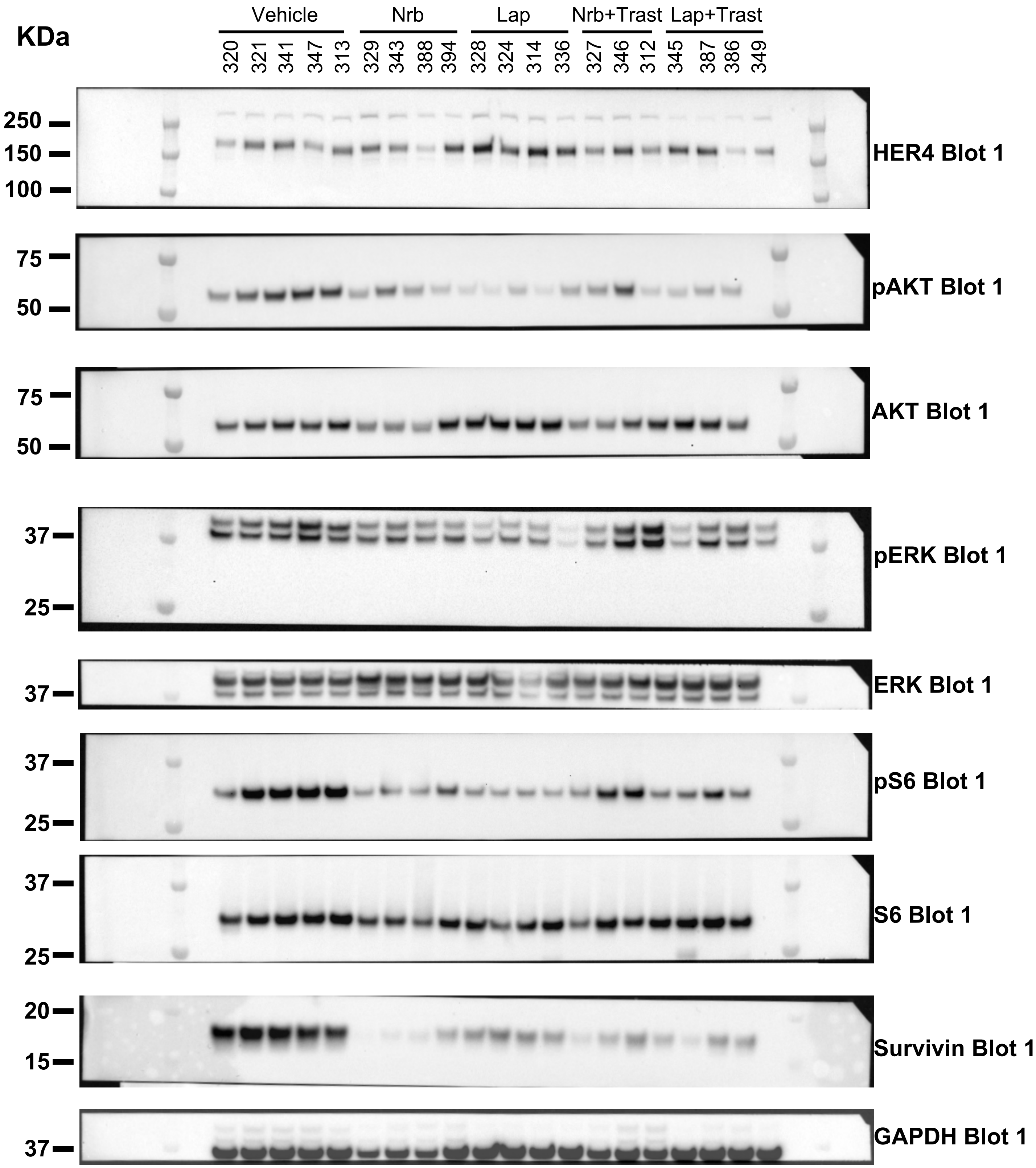

## Uncropped western blot images of Supplementary figure 8a

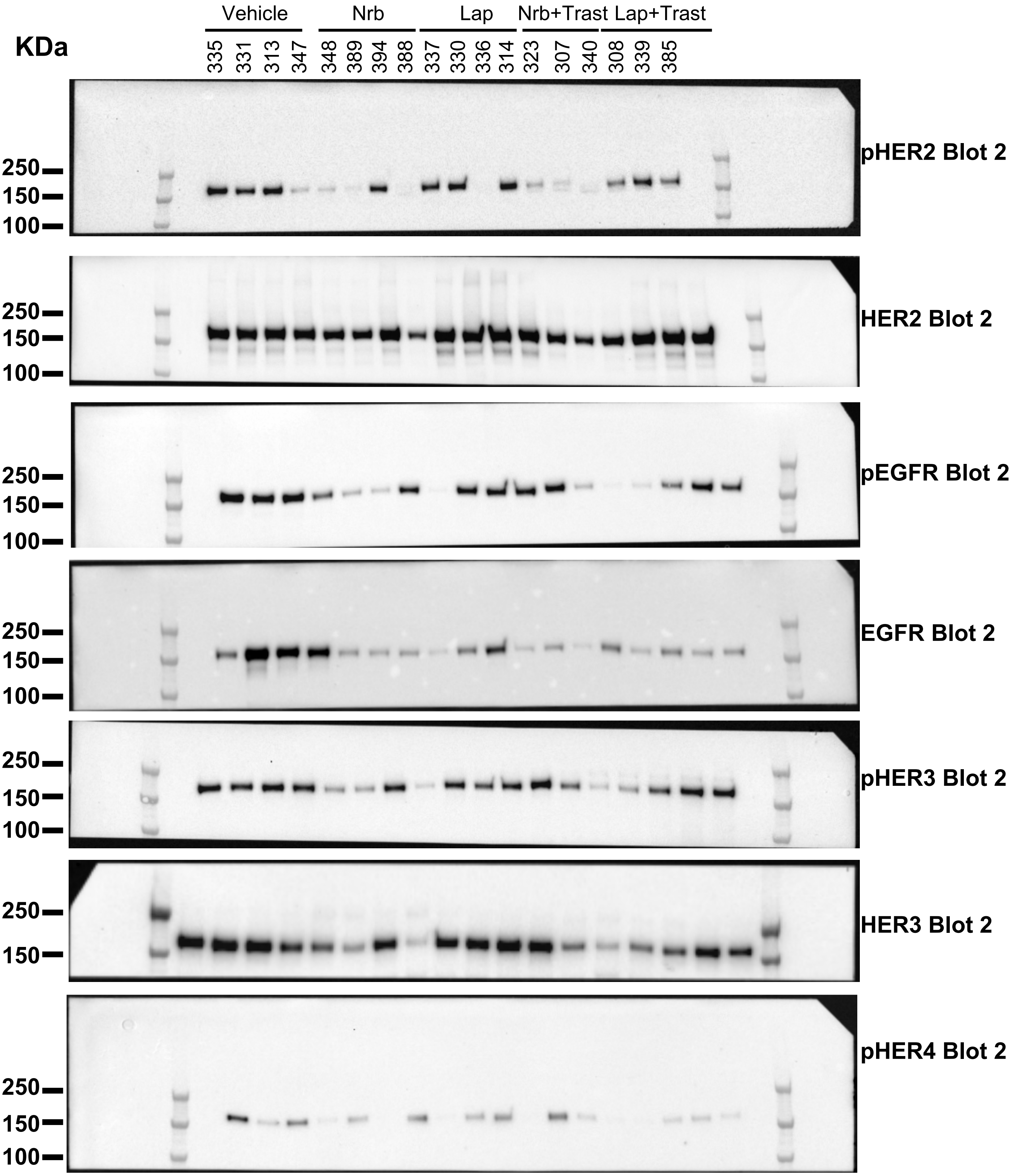

## Uncropped western blot images of Supplementary figure 8a

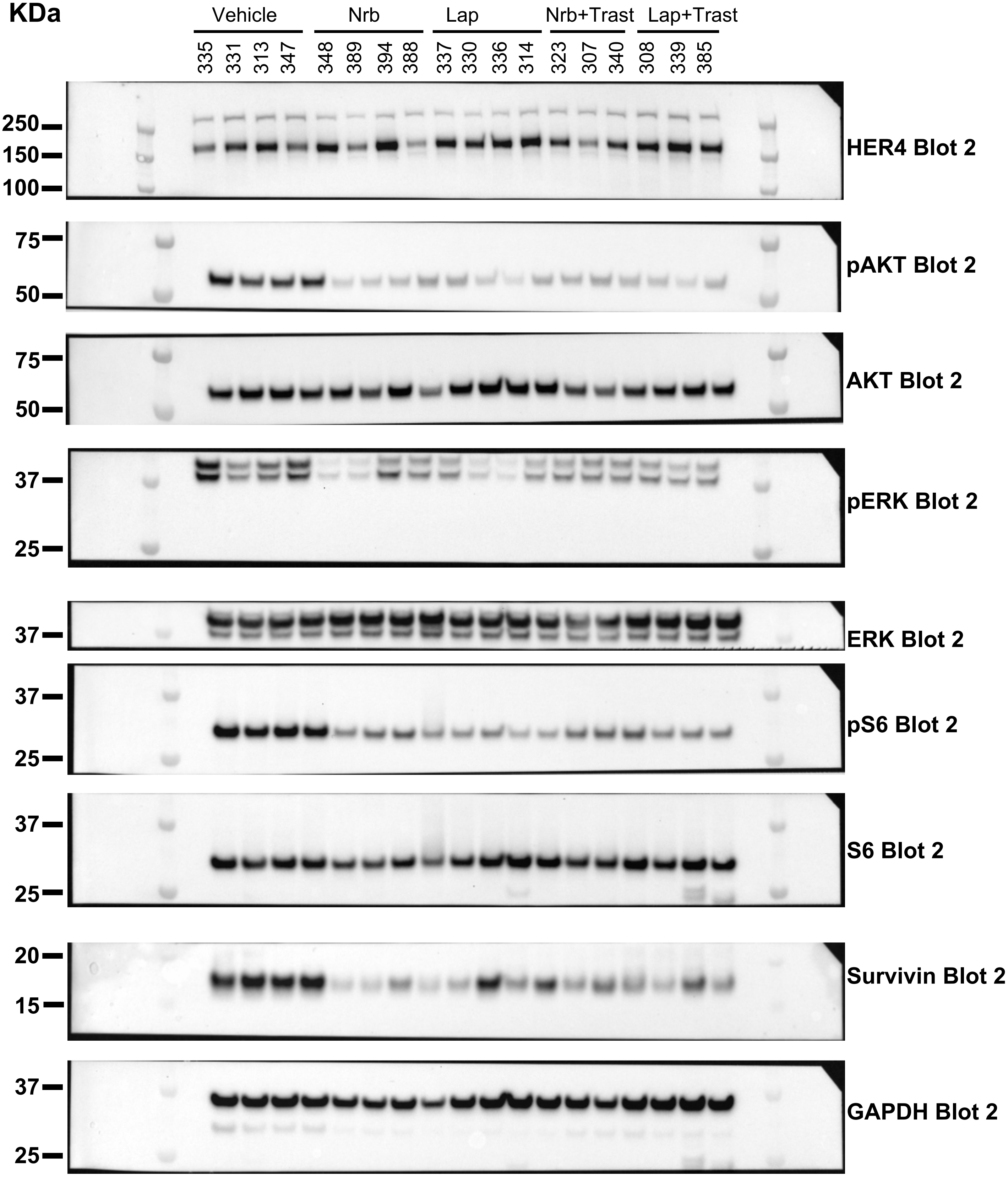

Supplement: Supplementary file 1 — Supplementary Information [file 41523_2021_274_MOESM1_ESM.pdf]
