# Supplementary material for: Neratinib plus trastuzumab is superior to pertuzumab plus trastuzumab in HER2-positive breast cancer xenograft models
Source: NPJ Breast Cancer. 2021 May 27;7:63. doi: 10.1038/s41523-021-00274-0 (PMC8159999; doi:10.1038/s41523-021-00274-0)
Supplement: Supplementary file 2 — Reporting Summary [file 41523_2021_274_MOESM2_ESM.pdf]

nature research

Corresponding author(s): Drs. Rachel Schiff and Jamunarani Veeraraghavan

Last updated by author(s): Apr 21, 2021

Reporting Summary

Nature Research wishes to improve the reproducibility of the work that we publish. This form provides structure for consistency and transparency in reporting. For further information on Nature Research policies, see our Editorial Policies and the Editorial Policy Checklist.

Statistics

For all statistical analyses, confirm that the following items are present in the figure legend, table legend, main text, or Methods section.

n/a	Confirmed	
☐	☒	The exact sample size (n) for each experimental group/condition, given as a discrete number and unit of measurement
☐	☒	A statement on whether measurements were taken from distinct samples or whether the same sample was measured repeatedly
☐	☒	The statistical test(s) used AND whether they are one- or two-sided<br>*Only common tests should be described solely by name; describe more complex techniques in the Methods section.*
☐	☒	A description of all covariates tested
☐	☒	A description of any assumptions or corrections, such as tests of normality and adjustment for multiple comparisons
☐	☒	A full description of the statistical parameters including central tendency (e.g. means) or other basic estimates (e.g. regression coefficient) AND variation (e.g. standard deviation) or associated estimates of uncertainty (e.g. confidence intervals)
☐	☒	For null hypothesis testing, the test statistic (e.g. F, t, r) with confidence intervals, effect sizes, degrees of freedom and P value noted<br>*Give P values as exact values whenever suitable.*
☒	☐	For Bayesian analysis, information on the choice of priors and Markov chain Monte Carlo settings
☒	☐	For hierarchical and complex designs, identification of the appropriate level for tests and full reporting of outcomes
☒	☐	Estimates of effect sizes (e.g. Cohen's d, Pearson's r), indicating how they were calculated

Our web collection on statistics for biologists contains articles on many of the points above.

Software and code

Policy information about availability of computer code

Data collection	The Image Lab™ software from Bio-Rad Laboratories, Inc. was used to generate images of western blots and to quantify them
Data analysis	Immunohistochemistry scores and western blot quantifications were analyzed by one-way or two-way ANOVA with Bonferroni multiple comparisons correction using GraphPad Prism V6.05 (CA, USA)

For manuscripts utilizing custom algorithms or software that are central to the research but not yet described in published literature, software must be made available to editors and reviewers. We strongly encourage code deposition in a community repository (e.g. GitHub). See the Nature Research guidelines for submitting code & software for further information.

Data

Policy information about availability of data

All manuscripts must include a data availability statement. This statement should provide the following information, where applicable:

- Accession codes, unique identifiers, or web links for publicly available datasets
- A list of figures that have associated raw data
- A description of any restrictions on data availability

The data generated and analyzed during this study are described in the following data record: 10.6084/m9.figshare.13284146. Datasets supporting Figures 1-4, Supplementary figures S2-S9 are publicly available in the figshare repository as part of the above data record. Uncropped western blot images supporting Figures 3-4, Supplementary figure 8 are provided in the supplementary file.

Field-specific reporting

Please select the one below that is the best fit for your research. If you are not sure, read the appropriate sections before making your selection.

☒ Life sciences ☐ Behavioural & social sciences ☐ Ecological, evolutionary & environmental sciences

For a reference copy of the document with all sections, see nature.com/documents/nr-reporting-summary-flat.pdf

Life sciences study design

All studies must disclose on these points even when the disclosure is negative.

Sample size

5-6 week old athymic nude mice or 4-5 week old SCID/Beige mice (Envigo, USA) were used for short-term (n=5-7 mice/arm; treated for 3.5 days) or long-term treatments (n=9-14 mice/arm)

Data exclusions

Tumor volumes from some mice in the BT474/AZ and BCM-3963 xenograft models were excluded from all analysis, and the exclusion criteria was pre-established. In both BT474/AZ and BCM-3963 xenograft models, data from mice that died very early in the experiment due to unknown health reasons were excluded from all analysis. In addition, in the BT474/AZ model, data from mice that accidentally received the wrong drug dosage were also excluded. Similarly, in the BCM-3963 model, data from mice that suffered health complications early on in the experiment due to accidental administration of drugs using gavage needles with a bigger gauge, which was inappropriate for the smaller SCID mice were excluded.
Data excluded from all analysis are provided in the following data record: 10.6084/m9.figshare.13284146.

Replication

Our findings of the differential efficacy of various HER2-targeted treatment regimens have been repeated using two different mouse breast cancer xenograft models. In addition, the efficacy of various agents have been tested at multiple levels, including anti-tumor efficacy as well as biomarkers. Furthermore, with regards to biomarker analysis, changes in the level and expression of key HER2 pathway-related, proliferative, and pro-apoptosis molecules have been confirmed using different means, including immunohistochemistry and western blot analysis. Additionally, the effect of different targeted agents on tumor cell proliferative activity have been confirmed using different methods and markers, including Ki67 and p-H3 levels using immunohistochemistry, and survivin by western blot.

Randomization

Mice bearing ~250 or 350 mm3 tumors were randomly assigned to different long-term and short-term treatment groups, respectively

Blinding

During the measurement of tumors in mice, the investigators were not blinded since the experimental procedure mandated grouping of mice randomized to each treatment group in separate cages with the specific treatment marked on each cage. However, with regards to biomarker analysis, the pathologist was blinded to treatment groups while scoring the immunohistochemistry slides.

Reporting for specific materials, systems and methods

We require information from authors about some types of materials, experimental systems and methods used in many studies. Here, indicate whether each material, system or method listed is relevant to your study. If you are not sure if a list item applies to your research, read the appropriate section before selecting a response.

Materials & experimental systems

n/a	Involved in the study	
☐	☒	Antibodies
☐	☒	Eukaryotic cell lines
☒	☐	Palaeontology and archaeology
☐	☒	Animals and other organisms
☒	☐	Human research participants
☒	☐	Clinical data
☒	☐	Dual use research of concern

Methods

n/a	Involved in the study	
☒	☐	ChIP-seq
☒	☐	Flow cytometry
☒	☐	MRI-based neuroimaging

Antibodies

Antibodies used

All the antibodies used in this study are listed in Supplementary table S1

Validation

In this study, all antibodies are used in applications validated by the manufacturer and as published before.
All antibodies purchased from cell signaling technology are validated by the manufacturer, as described in the manufacturer's website (https://www.cellsignal.com/about-us/our-approach-process/cst-antibody-validation-principles), using Hallmarks of Antibody Validation strategy described in Uhlen, et. al., ("A Proposal for Validation of Antibodies." Nature Methods (2016)).

For other antibodies, previous publications citing their use in applications recommended and validated by the manufacturer are listed below.
HER2 (Calbiochem, Cat # OP15): PMID 1978777
HER3 (Millipore, Cat # 05-390): PMID 25375092, PMID 24862757, and PMID 19894763.

	S6 (Santa Cruz Biotechnology, Cat # SC-74576): PMID 27993682, PMID 25605243, and PMID 25350163.<br>GAPDH (Santa Cruz Biotechnology, Cat # SC-365062): PMID 33619816, PMID 33545358, and PMID 33495297

Eukaryotic cell lines

Policy information about cell lines

Cell line source(s)	The BT474/AZ cell line was obtained from AstraZeneca (PMID 17470737)
Authentication	The BT474/AZ cell line was authenticated at the MD Anderson Characterized Cell Line Core Facility within 6 months of performing the experiments
Mycoplasma contamination	The BT474/AZ cell line was tested to be mycoplasma-free using the MycoAlert™ Mycoplasma Detection Kit (Lonza)
Commonly misidentified lines<br>(See ICLAC register)	This study did not use any commonly misidentified cell lines

Animals and other organisms

Policy information about studies involving animals; ARRIVE guidelines recommended for reporting animal research

Laboratory animals	5-6 week old female athymic nude mice, and 3-4 week old female SCID/Beige mice purchased from Envigo, USA were used in this study
Wild animals	This study did not involve use of wild animals
Field-collected samples	This study did not involve samples collected from the field
Ethics oversight	All animal studies were conducted in accordance with and approved by the Institutional Animal Care and Use Committee (IACUC) of Baylor College of Medicine

Note that full information on the approval of the study protocol must also be provided in the manuscript.
